# Proteomic Analysis in Morquio A Cells Treated with Immobilized Enzymatic Replacement Therapy on Nanostructured Lipid Systems

**DOI:** 10.3390/ijms20184610

**Published:** 2019-09-18

**Authors:** J. Víctor Álvarez, Susana B. Bravo, María García-Vence, María J. De Castro, Asteria Luzardo, Cristóbal Colón, Shunji Tomatsu, Francisco J. Otero-Espinar, María L. Couce

**Affiliations:** 1 Department of Pharmacology, Pharmacy and Pharmaceutical Technology, School of Pharmacy, Campus Vida, University of Santiago de Compostela, 15872 Santiago de Compostela, Spain; josevictor.Alvarezgonzalez@nemours.org; 2 Department of Forensic Sciences, Pathology, Gynecology and Obstetrics, Pediatrics, Neonatology Service, Department of Paediatrics, Hospital Clínico Universitario de Santiago de Compostela, Health Research Institute of Santiago de Compostela (IDIS), CIBERER, MetabERN, 15706 Santiago de Compostela, Spain; mj.decastrol@gmail.com (M.J.D.C.); cristobal.colon.mejeras@sergas.es (C.C.); 3 Skeletal Dysplasia Lab Nemours Biomedical Research Nemours/Alfred I. duPont Hospital for Children, 1600 Rockland Road, Wilmington, DE 19803, USA; 4 Proteomic Platform, Health Research Institute of Santiago de Compostela (IDIS), Hospital Clínico Universitario de Santiago de Compostela, 15706 Santiago de Compostea, Spain; sbbravo@gmail.com (S.B.B.); mariagarve@outlook.es (M.G.-V.); 5 Department of Pharmacology, Pharmacy and Pharmaceutical Technology, School of Sciences, Campus de Lugo, University of Santiago de Compostela, 27002 Lugo, Spain; asteriam.luzardo@usc.es; 6 Paraquasil Platform, Health Research Institute of Santiago de Compostela (IDIS), Hospital Clínico Universitario de Santiago de Compostela, 15706 Santiago de Compostela, Spain

**Keywords:** enzyme replacement therapy, lysosomal disorders, nanoparticles, proteomics

## Abstract

Morquio A syndrome, or mucopolysaccharidosis type IVA (MPS IVA), is a lysosomal storage disease due to mutations in the N-acetylgalactosamine-6-sulfatase (*GALNS*) gene. Systemic skeletal dysplasia and the related clinical features of MPS IVA are due to disruption of cartilage and its extracellular matrix, leading to an imbalance of growth. Enzyme replacement therapy (ERT) with recombinant human GALNS, alpha elosulfase, provides a systemic treatment. However, this therapy has a limited impact on skeletal dysplasia because the infused enzyme cannot penetrate cartilage and bone. Therefore, an alternative therapeutic approach to reach the cartilage is an unmet challenge. We have developed a new drug delivery system based on a nanostructure lipid carrier with the capacity to immobilize enzymes used for ERT and to target the lysosomes. This study aimed to assess the effect of the encapsulated enzyme in this new delivery system, using in vitro proteomic technology. We found a greater internalization of the enzyme carried by nanoparticles inside the cells and an improvement of cellular protein routes previously impaired by the disease, compared with conventional ERT. This is the first qualitative and quantitative proteomic assay that demonstrates the advantages of a new delivery system to improve the MPS IVA ERT.

## 1. Introduction

Morquio A syndrome, or mucopolysaccharidosis type IVA (MPS IVA, OMIM #253000), is an autosomal recessive disease, caused by mutations in the N-acetylgalactosamine-6-sulfatase (*GALNS*), gene which result in deficient activity of N-acetylgalactosamine-6-sulfatase (GALNS, E.C.3.1.6.4), an enzyme that degrades glycosaminoglycan (GAG) keratan sulate (KS) and chondroitin-6-sulfate (C6S) [1,2]. The widespread accumulation of GAG, primarily in chondrocytes, and its extracellular matrix, leads to progressive cellular damage and organ dysfunction in the bone and cartilage. The classical phenotype is characterized by systemic skeletal dysplasia, including a short stature and neck, cervical instability and spinal cord compression, tracheal obstruction, prominent chest, kyphoscoliosis, laxity of joints, hip dysplasia, knock knee, etc. [3]. In severe forms, respiratory failure is the primary cause of death during the second and third decade of life if untreated [4,5].

There are two treatments for MPS IVA patients in clinical practice: enzyme replacement therapy (ERT) and hematopoietic stem cell transplantation. ERT with elosulfase alpha, a recombinant human GALNS, is an established strategy for treating MPS IVA [1,6,7]. However, clinical trials have shown scarce improvement in growth and skeletal dysplasia [8,9,10,11]. In patients with MPS IVA, restrictive and obstructive lungs are due to an anatomical imbalance in growth (trachea and vessels grow while the spine and thoracic bones stop growing), leading to this life-threatening issue. ERT cannot affect bone deformity in the spine, ribs and sternum, and crowded thoracic cavity. Therefore, ERT may provide a limited impact on pulmonary function due to the remaining issue of skeletal dysplasia (restrictive lung). A recent long-term study of MPS IVA with ERT has shown that there was a global reduction in static spirometry values in all subjects with ERT, as well as cardiorespiratory function as assessed by 6MWT [12,13]. Similar to other ERTs for lysosomal storage diseases, elosulfase alpha has several pitfalls: (i) rapid clearance from circulation and a short half-life (2 min in mice; 35 min in humans) [10,12,14], (ii) limited penetration in the avascular cartilage, and (iii) immunological response against the infused enzyme [12,15,16]. Thus, therapies for resolving bone lesions remain an unmet need, and an alternative therapeutic approach is required to overcome such limitations. In this regard, one possible solution is a nanoparticular system that can protect the enzymes against their degradation or inactivation, minimize the associated immunological reactions, and increase cellular internalization to achieve maximum efficacy of the treatment [17]. Biodegradable nanoparticles of a different nature, such as polylactic-co-glycolic acid, have been used in ERT. Some studies have also shown the usefulness of solid lipid nanoparticles [8,12,15,16] to achieve the internalization of molecules, such as catalase, leuprolide, or insulin [18,19,20,21], to the cellular interior through their direct endocytosis towards lysosomes. One of the main limitations of these systems is their low capacity to immobilize and transport a protein into the lipid core. Some experiments have been carried out to absorb the protein on the surface of the lipid colloidal carriers, but in the case of enzymes, this approach can have drawbacks related to instability and the production of antibodies. Consequently, formulation design is a critical requirement to stabilize a high payload of the enzyme. We have recently developed a new drug delivery system to immobilize enzymes used for ERT and to target the lysosomes [nanostructure lipid carrier (NLC), patent registered number PCT/EP2019/068629].

Proteomics allows not only the identification but also the quantification of the overall proteins present in a cell, tissue, or organism in continuous change. One of the first proteomic approaches includes a one or two-dimensional polyacrylamide gel system using sodium dodecyl sulfate (1D SDS-PAGE and 2D SDS-PAGE) and provides a powerful tool to monitor protein purification and rapidly profile the global protein expression and posttranscriptional modifications [22]. Although 1D and 2D-PAGE are common proteomic approaches for high throughput screening of putative biomarkers in several disorders, particularly in animal studies [23,24], they have not been used in lysosomal diseases. On the other hand, mass spectrometry (MS) is a beneficial proteomic technology that allows proteomic pattern visualization and the identification of proteins [25,26] and their post-transcriptional modifications, such as phosphorylation, glycosylation, similar to 2D-Page approach [27]. In the last decade, MS has made significant progress by increasing the number of identified proteins [26]. Therefore, liquid chromatography (LC) coupled with MS is widely used for searching disease biomarkers [26] in samples such as biological fluids, tissues, cells, etc. [28,29]. 

Most quantitative methods involve labeling proteins with heavy and light stable-isotope pairs (SILAC, I-TRAQ). Other quantitation techniques eliminate labeling (label-free) and rely on advanced software analysis as a new technology named SWATH-MS (sequential window acquisition of all theoretical mass spectra) [30,31]. These methods measure the relative concentrations of peptide analytes within two or more samples. In contrast, absolute quantitation techniques use internal standard peptides that have been synthetically prepared for selected or multiple reaction monitoring (selected reaction monitoring or multiple reaction monitoring, respectively) analysis [25]. 

In general, disease states involve alterations in protein expression levels. Therefore, proteomics provides important biological information that contributes to the advance in our understanding of pathophysiological mechanisms and aid in the identification of novel biomarkers for different diseases [30,32,33]. Specific biomarkers identified from proteomics may be useful in diagnosis, prognosis, and as evaluators of treatment outcomes [34,35,36,37,38,39,40,41,42].

In this study, we have analyzed the effects of protein expression levels in the MPS IVA fibroblasts by providing the immobilized and free enzyme (GALNS) and by using qualitative and quantitative proteomic techniques.

## 2. Results

### 2.1. Qualitative Analysis Liquid Chromatography—Mass Spectrometry (DDA-LC-MS/MS)

By using DDA-LC-MS/MS, we identified 287 to 1623 proteins in untreated cells and 586 to 1817 proteins in fibroblasts treated with NLC + ERT or ERT alone (Table 1). 

**Figure 1:**
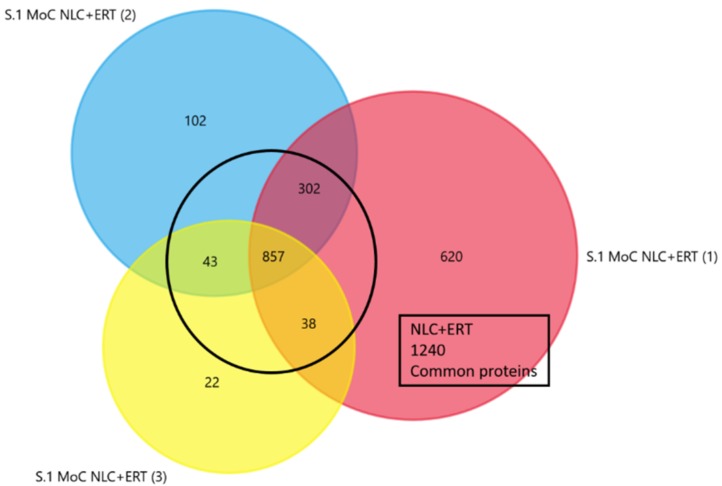
Venn diagram from the triplicates studies of proteins in Morquio A cells (Sample 1) after 24 h of NLC + ERT treatment. The common proteins identified in 2 or 3 of the replicates are shown.

To obtain a better representation of all the proteins identified in each treatment, we selected only those that were present in at least 2 of the replicates per treatment (Figure 1 and Supplementary Figure S1). 

**Figure 2:**
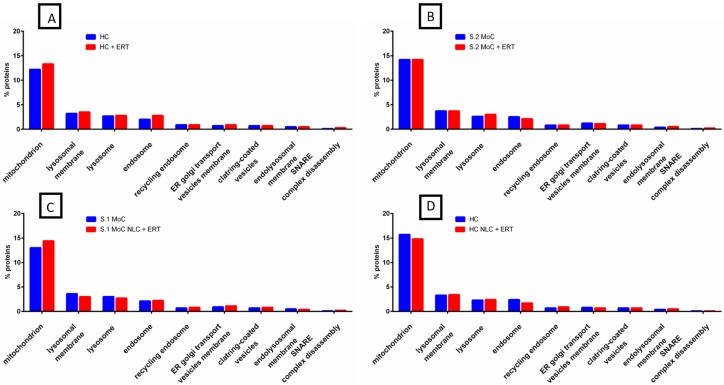
FunRich functional analysis results of expressed proteins from cellular components. (**A**) HC and HC + ERT. (**B**) UMoC (Sample 2) and MoC + ERT (Sample 2). (**C**) HC and HC with NLC + ERT. (**D**) UMoC (Sample 1) and MoC with NLC + ERT (Sample 1).

To analyze the origin of the identified proteins under no treatment, NLC + ERT, and ERT alone, we used FunRich analysis. The majority of proteins in all the samples were found to be sourced from four different cellular components: mitochondrion, lysosomal membrane, lysosome, and endosome (Figure 2A). In healthy cells (HC) with ERT (HC + ERT), we found an increase in a small percentage of proteins compared with untreated healthy cells (UHC), particularly for the mitochondrion and endosome. In the comparison between untreated Morquio A cells (UMoC) vs. Morquio A cells with ERT (MoC + ERT), no changes (or a minimal change) were found (Figure 2B and Supplementary Figure S2). When we compared HC vs. HC with NLC + ERT (HC NLC + ERT) (Figure 2C and Supplementary Figure S2), we found only a small decrease in the percentage of mitochondrion and endosome. When we compared UMoC vs. the MoC NLC + ERT (Figure 2D and Supplementary Figure S2), small differences in the percentage of proteins (a slight increase in mitochondrion and endosome and a slight decrease in lysosomal membrane and lysosome) were found.

**Figure 3:**
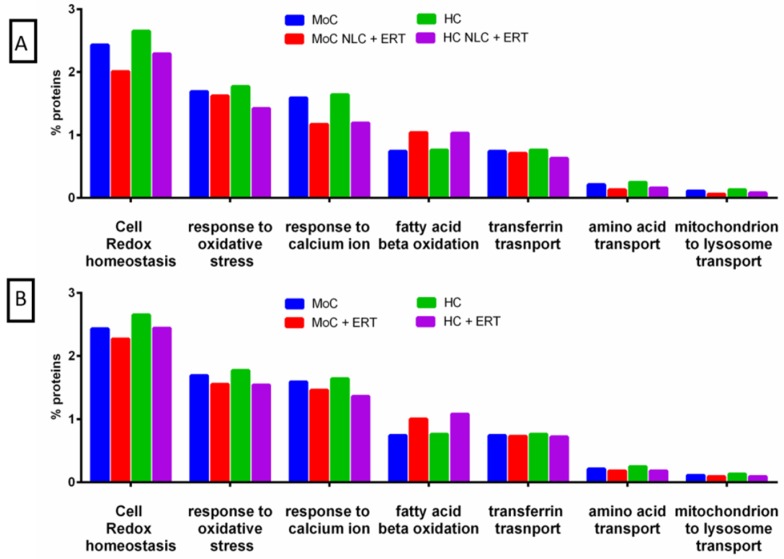
FunRich functional analysis results of the expressed proteins from the metabolic process of mitochondrion. (**A**) Comparison between untreated and treated for 24 h with NLC + ERT in HC and MoC. (**B**) Comparison between untreated and treated for 24 h with ERT alone in HC and MoC.

Regarding the biological process of the mitochondrion, we found changes in the proteins to be related to the interaction between the mitochondria and the lysosome (Figure 3). These changes were more prominent in the cells treated with NLC + ERT (Figure 3A) or ERT (Figure 3B). The analysis indicates that the expression level of proteins related to cell redox homeostasis, the response to oxidative stress, the response to calcium ion, transferrin transport, amino acid transport, and mitochondrion to lysosome transport were decreased in both treatments, while the expression levels of proteins related to fatty acid β-oxidation were increased. 

**Figure 4:**
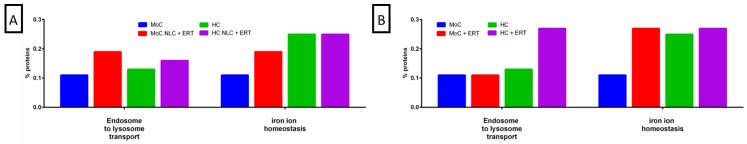
FunRich functional analysis results of expressed proteins from the metabolic process of the endosome. (**A**) comparison between untreated and treated for 24 h with NLC + ERT in HC and MoC; (**B**) comparison between untreated and treated for 24 h with ERT in HC and MoC.

When we analyzed the biological process of the endosome (Figure 4), the changes in the proteins involved in the endosome-to-lysosome transport and iron ion homeostasis were observed. We found an increase in endosome-to-lysosome transport in both HC and MoC with NLC + ERT (Figure 4A) but only in HC with ERT alone (Figure 4B). In the case of the iron ion homeostasis transport, we found a significant increase in MoC with both NLC + ERT and ERT alone (Figure 4A,B) and only a small increase in HC with ERT (Figure 4B).

**Figure 5:**
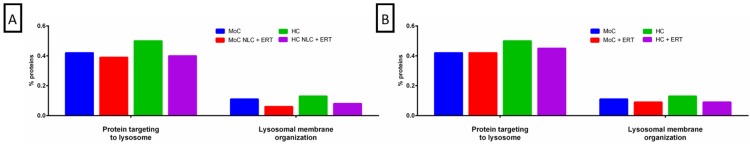
FunRich functional analysis results of expressed proteins from the metabolic process of the lysosomal membrane. (**A**) comparison between no treatment and 24 h treatment with NLC + ERT in HC and MoC (**B**) comparison between no treatment and 24 h treatment with ERT in HC and MoC.

In the lysosomal membrane (Figure 5), we observed a decrease of the expression level in the proteins related to targeting the lysosome and to the lysosomal membrane organization in NLC + ERT (Figure 5A). However, in ERT treatments, we only found a decrease in the lysosomal membrane (Figure 5B). Moreover, it should be noted that in HC, both HC alone or with treatment (NLC + ERT or ERT), we found that the targeted lysosome and lysosomal membrane organization protein levels decrease (Figure 5A,B).

**Figure 6:**
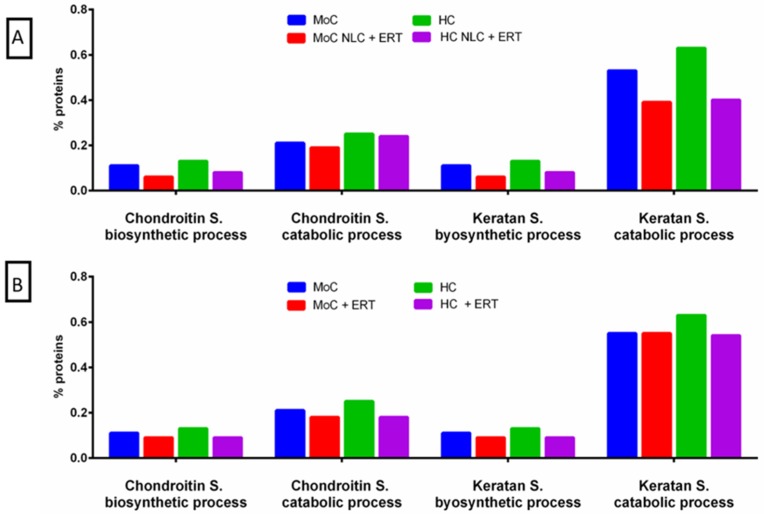
FunRich functional analysis results of the expressed proteins from the metabolic process of the lysosome. (**A**) comparison between no treatment and 24 h treatment with NLC + ERT in HC and MoC (**B**) comparison between no treatment and 24 h treatment with ERT in HC and MoC.

Related to the biological processing of proteins in the lysosome (Figure 6), we analyzed both the biosynthesis and the catabolism of C6S and KS. We observed that in cells treated with NLC + ERT (Figure 6A) or ERT (Figure 6B), the proteins related to these processes were decreased.

**Figure 7:**
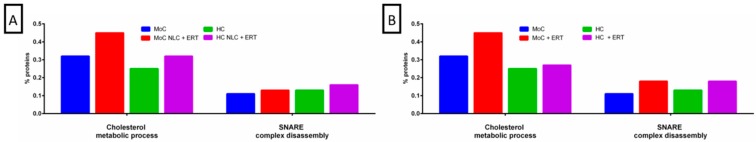
FunRich functional analysis results of expressed proteins from the metabolic process of the soluble N-ethylmaleimide-sensitive factor attachment protein receptor (SNARE) complex. (**A**) comparison between non-treatment and 24 h treatment with NLC+ ERT in HC and MoC (**B**) comparison between non-treatment and 24 h treatment with ERT in HC and MoC.

We also analyzed the biological processes of proteins in the soluble N-ethylmaleimide-sensitive factor attachment protein receptor (SNARE) complex (Figure 7), specifically looking for changes in the metabolic pathway of cholesterol and SNARE complex disassembly. The percentage of proteins involved in these processes was increased with both treatments. 

**Figure 8:**
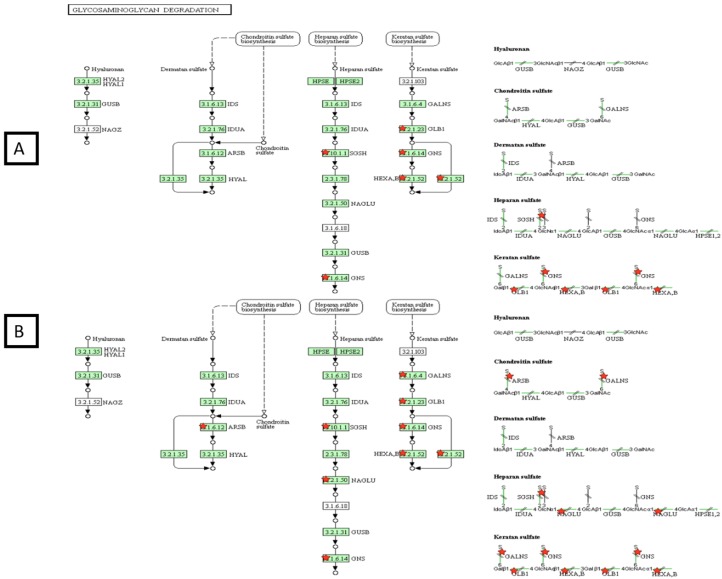
DAVID functional analysis of the glycosaminoglycan (GAG) degradation pathway in mucopolysaccharidosis type IVA (MPS IVA) fibroblasts without treatment (**A**) or NLC + ERT (**B**). Red stars correspond to proteins identified by the DDA-LC-MS/MS analysis. Green color of the boxes: sure confidence, black solid arrows: high confidence; black dotted arrows: moderate confidence.

With regards to the GAG degradation route (Figure 8), our results showed that, in UMoC only three of the four proteins implicated in the pathway were present. However, when we added NCL+ERT or ERT (data not shown), all the proteins appeared, thus completing the pathway and confirming that ERT provides an impact on the GAG degradation pathway.

**Figure 9:**
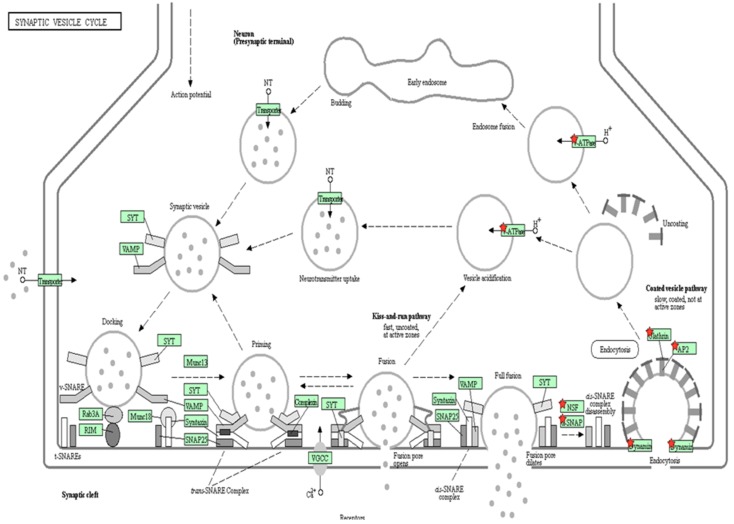
DAVID functional analysis of vesicle internalization. Red stars correspond to proteins identified by the DDA-LC-MS/MS analysis. Green color of the boxes: sure confidence, black solid arrows: high confidence; black dotted arrows: moderate confidence.

Accordingly, we also tested the vesicles’ internalization, which was previously described as mannose-6-phosphate receptor-dependent [43,44]. Our results indicate that NLC encapsulation of the drug induces internalization of vesicles in a different way through endocytosis, as we found proteins involved in the initial and final phases of endosome formation (Figure 2 and Figure 9).

**Figure 10:**
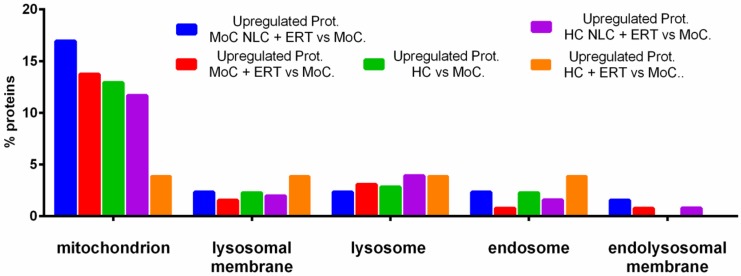
FunRich Functional analysis results of upregulated proteins showing the results related to protein internalization.

We obtained the quantitative measurement of proteins by another method (described in Section 2.2). We analyzed the same specimens with a new database (shown in Supplementary Table S1). In Figure 10, we show the percentage of upregulated proteins, comparing HC, UMoC, and MoC with NLC + ERT; MoC with ERT alone; HC with NLC + ERT; and HC with ERT alone in different organelles. The protein pattern expressions in the endolysosomal membrane are similar to those in the untreated affected cells and the HC. However, proteins related to the mitochondrion, lysosomal membrane, lysosome, and endosome were upregulated in the HC. Likewise, 17% of proteins in MoC treated with NLC + ERT were elevated in the mitochondrion when compared with the untreated cells. No significant difference was found in the remaining organelles.

### 2.2. Protein Quantification by SWATH-MS 

We employed this approach based on the use of a library in which quantification is performed to study deregulated proteins. To generate this library, the samples were pooled, and 4 µL of each sample was loaded into an DDA-LC-MS/MS system. A total of 1019 different proteins were identified (Supplementary Data 1). These proteins were selected because they had a global false discovery rate < 1% and an unused score (protein score) of > 1.2.

We found 11 upregulated proteins in UMoC, compared in ERT treated cells and, conversely, 43 upregulated proteins in MoC with ERT, compared in UMoC. These proteins had a *p* < 0.05 and a fold change (FC) > 1.2 (Table 2). 

In the comparison between UMoC vs. MoC with NLC + ERT, we found 14 upregulated proteins and, inversely, 63 downregulated proteins (Table 3).

We found 36 common upregulated proteins in MoC with NLC + ERT and ERT (Table 2 and Table 3 and Supplementary Table S1), of which 19 were located at the cytoplasm, 10 at the mitochondrion membrane, and 4 are secreted or located in the lysosome/endosome (Supplementary Table S1 and Figure 10). SWATH-MS quantitative analysis led to a similar result, showing again how the NLC + ERT increases the internalization of proteins mediated by vesicle transport (Figure 9 shows a qualitative test and Figure 10 shows quantitative assays). The total number of upregulated proteins are 66, of which 15 used any treatment; this number was 31 if we used ERT and 20 if we used NLC + ERT (Supplementary Figure S3).

## 3. Discussion

This is the first study that evaluates the effects of free and NLC encapsulated enzymes in MoC by using two proteomic approaches: a qualitative approach and a quantitative approach. Sleat et al. reported that proteomics approaches can determine the etiology of lysosomal storage disease by analyzing human brain samples [45]. The aim of our study was to assess the protein expression patterns in HC and MoC treated with free ERT and NLC encapsulated ERT, to evaluate the potential advantages of this novel therapeutic approach. 

Recent studies have demonstrated that lysosomes have more functions than those initially described by DeDuve and Novikoff in the last century [29,46,47] because the lysosomes interact with mitochondria and the endoplasmic reticulum and play major roles in the cytoplasm. Further investigations allowed a comprehensive understanding of the relation between these organelles and demonstrated their role in the regulation of amino acids and lipid metabolism, where Ca^2+^ ion is the key to complete these functions [48,49]. Moreover, a mismatch in the oxidation-reduction of the mitochondria can also be explained when alterations in the lysosomes occur. The proteins involved in the mitochondria–lysosome relation are the most important in our study because they are expected to be deregulated in the lysosomal diseases. Therefore, our interest is to assess their alterations in fibroblasts with MPS IVA and describe how the protein expression levels are modified when adding the free enzyme employed in the clinical practice alone or the encapsulated enzyme in our new delivery system. 

We identified the changes in the cellular component of the expressed proteins related to the mitochondrion and the interaction between the mitochondria and the lysosome of the cells treated with NLC + ERT vs. ERT. These findings are consistent with those described by Todkar et al. [50]. They show the physical and functional interaction between mitochondria and lysosomes, suggesting that this crosstalk plays a major role in metabolic regulation. It should be highlighted that the effects of the disease at the cellular level are reversed to a greater extent with the encapsulated ERT. NLC + ERT may achieve a greater effect with lower doses when compared to free ERT since it accomplishes a more efficient internalization in the lysosomes, thereby improving protein expression in pathological cells, as we can see in Figure 10. We also found the decrease of proteins to be related to redox homeostasis, the response to oxidative stress, the response to calcium ion, transferrin transport, amino acid transport, and mitochondrion to lysosome transport, while proteins related to fatty acid beta-oxidation were increased. These relationships demonstrate that our treatment can restore the lysosome and mitochondria interaction lacking in the Morquio A disease. These changes indicate that the new delivery system increases the drug’s effect. Another important factor to take into consideration is that transferrin’s function plays a very important role in the iron (Fe) ion transport to the mitochondria, which is necessary for endosome formation [50,51]. 

It is known that in mucopolysaccharidoses, a loss of the lysosome–endosome junction and endosomal accumulation occurs [52,53]. We found an increase in the percentages of proteins involved in the endosome to lysosome transport and iron ion homeostasis. This result indicates that our treatment reverts the endosome disruption due to the greater transport transport to the endosome, as iron is mainly found in endosome membranes, as we show in Figure 4. Moreover, we observed a decrease in the proteins targeting the lysosome, which indicates a lower accumulation of proteins that constitute the lysosome in both NLC + ERT and ERT treatments. In the current study, we have verified the decrease in proteins related to lysosomes when applying ERT or NLC + ERT. This may indicate a restored lysosome-Golgi-mitochondria-endosome interaction, which plays a major role in the trafficking process. On the other hand, when analyzing C6S and KS metabolism, we observed that in both treatments, the percentage of the proteins involved in this process were decreased, though this phenomenon was more prominent in NLC + ERT. We postulate that this occurs due to the reconstruction of the degradation pathway of both C6S and KS. 

Other lysosomal functions described by Novikoff et al. are the autophagy process proteins, such as SNARE, which are crucial for the endosome–lysosome fusion in the final phase of autophagy [52]. In our study, the percentage of proteins related to SNARE was increased in both treatments, which may indicate increased lysosome–endosome fusion [52,54,55]. Moreover, we also observed changes in cholesterol metabolism, finding it to increase in both treatments. Cholesterol induces SNARE proteins to free themselves from the endosomes, thus facilitating the lysosome–endosome union.

This study also demonstrates that the internalization process in NLC + ERT is mediated by endosomes via clathrin receptors, as we show in Figure 9, and it is easier for the proposed nanoparticle drug to target lysosomes than ERT. This is because elosulfase alpha is internalized via the mannose 6-phosphate receptors, which are saturable. At a certain point, high enzyme concentrations may block the enzyme’s internalization via saturation of the membrane receptor [43,44]. On the other hand, we performed a SWATH-MS quantitative assay and found 66 upregulated proteins, 15 of which were upregulated in both ERT and NLC + ERT, while the other 20 were upregulated only in NLC + ERT. In addition, between the upregulated proteins in the MoC treated with NLC + ERT, we should highlight the Collagen type VI alpha-3 chain protein, which is a biomarker of collagen function. This finding suggests that we are improving the expression of proteins related to the connective tissue in Morquio cells, making it more similar to healthy cells [56,57]. These results indicate the different processes underlying the function and internalization of free ERT vs. NLC + ERT. 

Finally, the main limitation of this study is the low number of samples evaluated. This is because MPS IVA is a rare disease with a low number of affected people. These findings should be confirmed in a larger sample of patients.

## 4. Materials and Methods 

### 4.1. The Work Scheme of This Study

**Figure 11:**
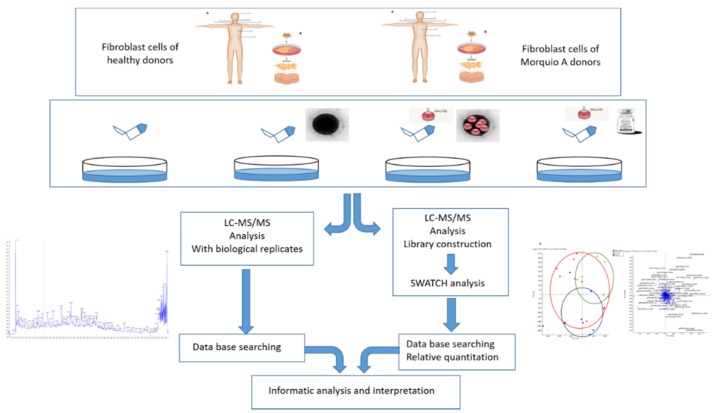
Graphical abstract showing the workflow. Fibroblasts obtained from healthy subjects and morquio A patients were cultured in vitro. After 24 h of treatment, cells were lysed and proteomic analyses were done. On the left, the identified proteins expressed by the cells (qualitative analysis) are shown. On the right, the amount of specific proteins expressed by the cells (quantitative analysis) are shown. Once this two different proteomics assessments were performed, an informatics analysis was made in order to obtain more information (localization, pathways, interactions…) about the identified/quantified proteins.

Graphical abstract shows the workflow of the qualitative (DDA-LC-MS/MS) and quantitative (SWATH-MS) proteins identification in the fibroblasts from healthy vs. Morquio patients (Figure 11).

### 4.2. Sample Resources

Fibroblast cells from 4 different MoC samples and 1 HC sample were obtained for the study through international biobanks (Telethon network biogenetic biobank, http://dppm.galini.org/biobank/). The mean age of the patients with MPSIVA was 25.2 years old (range 23–30 years). This group included two males and two females. The HC was a male of 25 years of age. The study was approved (22 May 2017) by the local Ethics Committee of Santiago-Lugo (Reference number 2017/298). Additional information about the characteristics of the samples are shown in Table 4.

### 4.3. Cell Cultures in Fibroblasts

Fibroblasts were obtained from the anterior forearm tissue. Samples were cultured at 37 °C (HERA cell 150, Thermo Fisher Scientific, Lancashire, UK) and 5% CO_2_ in a modified all GIBCO McCoy 5A medium supplemented with 10% FBS, 1% penicillin-streptomycin, and 0.25% trypsin-EDTA 1X. After the cells grew out of the tissue fragments and reached a 60% of confluence, the treatment was added (100 ng of elosulfase alpha for each treatment: ERT and NLC + ERT) (Table 5). The fibroblasts were then detached and removed by employing a sterile phosphate buffer (Sigma, St Louis, MO, USA) and trypsin. The cells were then washed with a PBS solution.

### 4.4. Materials and Preparation of NLC

Elosulfase alpha (Vimizim^®^) was provided by Biomarin (San Francisco, CA, USA). Block copolymers (Kolliphor^®^ P407 and Kolliphor P188) and D-α-tocopherol polyethylene glycol 1000 succinate were purchased from Sigma Aldrich (St Louis, MO, USA). Glycerildibehenate (Compritol 888 ATO) was obtained from Gatefossé (Lyon, France). Trimyristin (Dynasan 114) and triestearin (Dynasan 118) were purchased from (KemCareLondon, UK). Cholesterol lanolin was obtained from Fluka (Munich, Germany) while olive oil, soy lecithin, and caprylic/capric triglyceride (Miglyol 812N) were from Acofarma SCL (Barcelona, Spain). The water used was ultrapure (milli Q), and all other chemicals were of analytical grade. 

NLC was prepared using the fast-double emulsification (O/W/O) and low temperature-solidification techniques. Briefly, the lipid forming-components of the coating were dissolved in dichloromethane. Elosulfase alpha dissolution was added to the aqueous phase with the hydrophilic surfactant (Kolliphor^®^ P407, (was purchased from Sigma Aldrich. St Louis, MO, USA)) and homogenized using ultrasound (Branson 450, Danbury, CT, USA) to obtain the first emulsion. To this resulting O/W emulsion, a second water phase was added. This phase was composed of D-α tocopherol polyethylene glycol 1000 in PBS (as PEGylant agent), and the obtained mixture was homogenized again using ultrasound. Kolliphor^®^ 188 solution in PBS was added to remove any aggregates formed during preparation, and NLC formulations were filtered through polyamide filters (0.45 µm). To isolate the NLC, ultracentrifuge (Beckman L8-70M, Ramsey, MN, USA) was used (35 min; 35,000rpm; 15 °C). Finally, NLC formulations were lyophilized from an aqueous suspension with different concentrations (5; 10; 20%) of several cryoprotectants (glucose; mannitol; trehalose; sucrose) for 24–48 h (Telstar Lyoquest-85 Bristol, PA, USA). 

### 4.5. Protein Extraction

Cells were sonicated, to break the membrane, and centrifuged for 10 min at 10,000 rpm at 4 °C. Protein extracts found in the supernatant were recovered and subsequently frozen at −20 °C.

### 4.6. Enzyme Activity Test

The enzyme activity of GALNS in the samples was analysed via the technique used for the diagnosis of the Morquio A disease [58]. The values are shown in Table 6 (normal values 1.8–20.0 nM/h/mg).

### 4.7. Proteomic Analysis 

#### 4.7.1. In Gel Protein Digestion

To ensure global and quantitative (by SWATH-MS) protein identification, an equal amount of proteins from the treated patients and controls were loaded on a 10% SDS-PAGE gel. The run was stopped as soon as the front had penetrated 3 mm into the resolving gel [59,60]. The protein band was detected by Sypro-Ruby fluorescent staining (Lonza, Basel, Switzerland), excised, and processed for in-gel and manual tryptic digestion, as described [61]. Gel pieces were reduced with 10 mM dithiothreitol (Sigma-Aldrich, St. Louis, MO, USA) in 50 mM ammonium bicarbonate (Sigma-Aldrich, St. Louis, MO, USA) and alkylated with 55 mM iodoacetamide (Sigma-Aldrich, St. Louis, MO, USA) in 50 mM ammonium bicarbonate. Then, the gel pieces were rinsed with 50 mM ammonium bicarbonate in 50% methanol (HPLC grade, Scharlau, Barcelona, Spain), dehydrated via the addition of acetonitrile (HPLC grade, Scharlau, Barcelona, Spain), and dried in a SpeedVac. Modified porcine trypsin (Promega, Madison, WI, USA) was added to the dry gel pieces at a final concentration of 20 ng/μL in 20 mM ammonium bicarbonate, incubating them at 37 °C for 16 h. The peptides were extracted thrice by 20 min incubation in 40 μL of 60% acetonitrile in 0.5% formic acid. The resulting peptide extracts were pooled, concentrated in a SpeedVac (TermoFisher Scientific: Haysham, Lancashire), and stored at −20 °C.

#### 4.7.2. Mass Spectrometric Analysis (DDA acquisition)

Digested peptides were separated using Reverse Phase Chromatography. The gradient was created using a micro liquid chromatography system (Eksigent Technologies nanoLC 400, ABSciex, (Warrington, Cheshire, UK) coupled to high-speed Triple TOF 6600 mass spectrometer (ABSciex, Foster City, CA, USA), with a micro flow source, as described previously [26,27]. The chosen analytical column was a silica-based reversed-phase column Eksigent C18CL 150 × 0.30 mm, 3 µm particle size, and 120 Å pore size (Eksigent, ABSciex, Woodlands Central Indus. Estate, Singapore). The trap column was a YMC-TRIART C18 (YMC Technologies, Teknokroma, Barcelona, Spain) with a 3 µm particle size and 120 Å pore size, switched on-line with the analytical column. The loading pump delivered a solution of 0.1% formic acid in water at 10 µL/min. The micro-pump generated a flow-rate of 5 µL/min and operated under gradient elution conditions, using 0.1% formic acid in the water as mobile phase A, and 0.1% formic acid in acetonitrile as mobile phase B. The peptides were separated using a 90 min gradient ranging from 2% to 90% of mobile phase B (mobile phase A: 2% acetonitrile, 0.1% formic acid; mobile phase B: 100% acetonitrile, 0.1% formic acid). The injection volume was 4 µL (more or less 4 µg of protein). 

Data acquisition was performed in a TripleTOF 6600 System (ABSciex, Foster City, CA, USA) using a data dependent workflow. Source and interface conditions were the following: ion spray voltage floating (ISVF) 5500 V, curtain gas (CUR) 25, collision energy (CE) 10, and ion source gas 1 (GS1) 25. The instruments were operated with the Analyst TF 1.7.1 software (ABSciex, Woodlands Central Indus. Estate, Singapore). The switching criteria were set to ions greater than the mass to charge ratio (*m*/*z*) 350 and smaller than *m*/*z* 1400, with a charge state of 2–5, a mass tolerance of 250 ppm, and an abundance threshold of more than 200 counts (cps). Former target ions were excluded for 15 s. The instruments were automatically calibrated every 4 h using external calibrant tryptic peptides from PepCalMix (Sciex, Warrington, Cheshire, UK).

#### 4.7.3. Data Analysis

After the MS/MS analysis, the data files were processed using the ProteinPilotTM 5.0.1 software from ABSciex (Woodlands Central Indus Estate, Singapore), which uses the algorithm ParagonTM for database searching and ProgroupTM for data grouping. Data were searched using a Human-specific Uniprot database (www.uniprot.org). The false discovery rate was determined using a non-linear fitting method displaying only those results that reported a 1% Global false discovery rate or better [28].

Functional analysis was performed by different open access software. We used **FunRich** (functional enrichment analysis tool) for functional enrichment and interaction network analysis (Available online: http://funrich.org/index.html). For statistics, we used FunRich (hypergeometric test), Bonferroni [29,30], network construction, and clustering. We also used DAVID (Available online: https://david.ncifcrf.gov/) for GAG degradation pathways and functional analysis of the vesicle internalization.

### 4.8. Protein Quantification by SWATH-MS 

#### 4.8.1. Creation of the Spectral Library

To build the MS/MS spectral libraries, the peptide solutions were analyzed by a shotgun data-dependent acquisition (DDA) approach using micro-LC-MS/MS (ABSciex, Redwood City, CA, USA), as described previously by us and other authors [27,46,47,62]. To obtain a good representation of the peptides and proteins present in all samples, pooled vials of the samples from each group were prepared using equal mixtures of the original samples. A total of 4 μL from each pool was separated into a micro-LC system Ekspert nLC425 (Eksigen, Dublin, CA, USA) using an Eksigent C18 150 × 0.30 mm, with 3 mm particle size and 120 Å pore size (Eksigent, ABSciex), at a flow rate of 5 mL/min. Water and ACN, both containing 0.1% formic acid, were used as solvents A and B, respectively. The gradient run consisted of 5% to 95% B for 30 min, 5 min at 90% B, and, finally, 5 min at 5% B for column equilibration, for a total run time of 40 min. As the peptides eluted, they were directly injected into a hybrid quadrupole-TOF mass spectrometer Triple TOF 6600 (ABSciex, Redwood City, CA, USA) operated with a data-dependent acquisition system in positive ion mode. A Micro source (ABSciex) was used for the interface between microLC and MS, with an application of 2600 V voltage. The acquisition mode consisted of a 250 ms survey MS scan from 400 to 1250 *m*/*z*, followed by an MS/MS scan from 100 to 1500 *m*/*z* (25 ms acquisition time) of the top 65 precursor ions from the survey scan, for a total cycle time of 2.8 s. The fragmented precursors were added to a dynamic exclusion list for 15 s. Any singly charged ions were excluded from the MS/MS analysis.

The peptide and protein identifications were performed using Protein Pilot software (version 5.0.1, ABSciex), and the data were searched using a Human-specific Uniprot database, specifying iodoacetamide as the Cys alkylation. The false discovery rate (FDR) was set to 1 for both peptides and proteins. The MS/MS spectra of the identified peptides were then used to generate the spectral library for the SWATH-MS peak extraction using the add-in for the PeakView Software (version 2.2, ABSciex) MS/MSALL with the SWATH-MS Acquisition MicroApp (version 2.0, ABSciex). Peptides with a confidence score above 99% (as obtained from Protein Pilot database search) were included in the spectral library).

#### 4.8.2. Relative Quantification by SWATH-MS Acquisition 

SWATH–MS acquisition was performed on a TripleTOF^®^ 6600 LC-MS/MS system (ABSciex). Each sample (4 μL) was analyzed using the LC-MS/MS equipment (Sciex, Warrington, Cheshire, UK) and the LC gradient described above for building the spectral library, but instead used the SWATH-MS acquisition method. The method consisted of repeating a cycle that consisted of the acquisition of 65 TOF MS/MS scans (400 to 1500 *m*/*z*, high sensitivity mode, 50 ms acquisition time) of overlapping sequential precursor isolation windows of variable widths (1 *m*/*z* overlap), covering the 400 to 1250 *m*/*z* mass range with a previous TOF MS scan (400 to 1500 *m*/*z*, 50 ms acquisition time) for each cycle. The total cycle time was 6.3 s. For each sample set, the width of the 65 variable windows was optimized according to the ion density found in the DDA runs using a SWATH-MS variable window calculator worksheet from Sciex.

#### 4.8.3. Data Analysis 

The targeted data extraction of the fragment ion chromatogram traces from the SWATH-MS runs was performed by Peak View (version 2.2, ABSciex) using the SWATH-MS Acquisition Micro App (version 2.0, Sciex, Warrington, Cheshire, UK). This application processed the data using the spectral library created from the shotgun data. Up to ten peptides per protein and seven fragments per peptide were selected, based on signal intensity. Any shared and modified peptides were excluded from the processing. Five-minute windows and 30 ppm widths were used to extract the ion chromatograms. SWATH-MS quantization was attempted for all proteins in the ion library that were identified by Protein Pilot with an FDR below 1%. The retention times from the peptides that were selected for each protein were realigned in each run, according to the iRT peptides that spiked in each sample, and eluted along the whole-time axis. The extracted ion chromatograms were then generated for each selected fragment ion. The peak areas for the peptides were obtained by summing the peak areas from the corresponding fragment ions. Peak View computed an FDR and a score for each assigned peptide according to the chromatographic and spectra components. Only peptides with an FDR below 5% were used for protein quantization. Protein quantization was calculated by adding the peak areas of the corresponding peptides. 

The integrated peak areas were directly exported to the Marker View software (ABSciex) for relative quantitative analysis. The export generated three files containing quantitative information about individual ions, the summed intensity of the different ions for a particular peptide, and the summed intensity of different peptides for a particular protein. Marker View was used for analysis of the SWATH-MS data reported in other proteomics studies [34,62,63,64] because of its data-independent method of quantization. Marker View uses processing algorithms that accurately find chromatographic and spectral peaks direct from the raw SWATH-MS data. Data alignment by Marker View compensated for minor variations in both the mass and retention time values, ensuring that identical compounds in different samples were accurately compared to one another. To control for possible uneven sample loss across the different samples during the sample preparation process, we performed a global normalization based on the total sum of all the peak areas extracted from all the peptides and transitions across the replicates of each sample [65]. An unsupervised multivariate statistical analysis using principal component analysis (PCA) was performed to compare the data across the samples. The average MS peak area of each protein was derived from the replicates of the SWATH-MS of each sample followed by a Student’s *t*-test analysis using the Marker View software for comparison among the samples based on the averaged area sums of all the transitions derived for each protein. The *t*-test indicated how well each variable distinguished the two groups, reported as a *p*-value. For each library, each set of differentially expressed proteins (*p*-value *<* 0.05) with 1.2 up-regulated or down-regulated proteins was selected.

## 5. Conclusions

We have demonstrated that our new delivery system, through a nanostructure lipid carrier, can induce changes in proteins that are involved in the disease. This system used a different internalization pathway (not saturable) that seems to induce better access to the lysosome. Using a low enzyme dosage, we obtained excellent C6S and KS degradation, greater than with the free enzyme. Therefore, this system can improve the lysosome-endosome-mitochondria machine in the MoC.

## Figures and Tables

**Table 1:** The number of proteins identified in the qualitative (DDA-LC-MSMS) assay.

	Number of Proteins. 1st Analysis			Number of Proteins. 2nd Analysis			Number of Proteins. 3rd Analysis		
Samples	No Treat	NLC + ERT	ERT	No Treat	NLC + ERT	ERT	No Treat	NLC + ERT	ERT
HC	683	1395	1612	1561	1668	919	737		1183
S.1 MoC	730	960		923	1304		1623	1817	
S.2 MoC	1220		1255	832		1022	793		1078
S.3 MoC	793	1658		1251	1594		1419	1104	
S.4 MoC	611		1445	287		870	1403		586

Note: All the assays were made in triplicate. HC: healthy cells; treat: treatment; NLC: nanostructure lipid carrier; LC: lipid chromatography; ERT: Enzyme replacement therapy; MoC: Morquio A cells; S.1: Sample 1.

**Table 2:** Quantitative study of proteins in UMoC vs. MoC with ERT (A) and vice versa (B).

	**A. Upregulated Proteins in UMoC, Compared in MoC with ERT**		
**Protein**	**Group**	***p*-Value**	**FC**
P29692	Elongation factor 1-delta	0.0341	2.1053
P54727	UV excision repair protein RAD23 homolog B	0.0340	1.9325
P16401	Histone H1.5	0.0357	1.8159
P26373	60S ribosomal protein L13	0.0449	1.8140
O15143	Actin-related protein 2/3 complex subunit 1B	0.0329	1.7815
P31942	Heterogeneous nuclear ribonucleoprotein H3	0.0234	1.6284
P07437	Tubulin beta chain	0.0361	1.6138
Q07020 Q9Y4L1	60S ribosomal protein L18 Hypoxia up-regulated protein 1	0.0477 0.0104	1.4902 1.4313
P13674	Prolyl 4-hydroxylase subunit alpha-1	0.0299	1.3145
P68104	Elongation factor 1-alpha 1	0.0342	1.2451
**B. Upregulated Proteins in MoC with ERT, Compared in UMoC**			
**Protein**	**Group**	***p*-Value**	**FC**
P07237	Protein disulfide-isomerase	0.0408	1.2058
P78417	Glutathione S-transferase omega-1	0.0425	1.2224
O00299	Chloride intracellular channel protein 1	0.0195	1.2303
P23526	Adenosylhomocysteinase	0.0443	1.2358
Q96S97	Myeloid-associated differentiation marker	0.0496	1.2446
P62258	14-3-3 protein epsilon	0.0079	1.2498
P16152	Carbonyl reductase [NADPH] 1	0.0199	1.2521
P32119	Peroxiredoxin-2	0.0385	1.2540
P00387	NADH-cytochrome b5 reductase 3	0.0087	1.2629
P17655	Calpain-2 catalytic subunit	0.0287	1.2708
P61981	14-3-3 Protein gamma	0.0482	1.2743
P60900	Proteasome subunit alpha type-6	0.0270	1.2756
O43707	Alpha-actinin-4	0.0309	1.2781
P27824	Calnexin	0.0254	1.2798
P11413	Glucose-6-phosphate 1-dehydrogenase	0.0215	1.2906
P08133	Annexin A6	0.0055	1.2937
P12814	Alpha-actinin-1	0.0146	1.3017
P22314	Ubiquitin-like modifier-activating enzyme 1	0.0015	1.3163
P54578	Ubiquitin carboxyl-terminal hydrolase 14	0.0386	1.3214
P49721	Proteasome subunit beta type-2	0.0414	1.3568
Q96AG4	Leucine-rich repeat-containing protein 59	0.0085	1.3642
P67812	Signal peptidase complex catalytic subunit SEC11A	0.0153	1.3671
Q15404	Ras suppressor protein 1	0.0172	1.3808
P07996	Thrombospondin-1	0.0379	1.3992
O00629	Importin subunit alpha-3	0.0386	1.4218
Q9BS26	Endoplasmic reticulum resident protein 44	0.0170	1.4754
Q99798	Aconitate hydratase, mitocondrial	0.0357	1.4799
P60953	Cell division control protein 42 homolog	0.0215	1.4806
Q9Y3I0	tRNA-splicing ligase RtcB homolog	0.0506	1.4807
P2484	Myosin regulatory light polypeptide 9	0.0344	1.5135
Q15758	Neutral amino acid transporter B(0)	0.0076	1.5970
Q13724	Mannosyl-oligosaccharide glucosidase	0.0351	1.6011
P08727	Keratin, type I cytoskeletal 19	0.0315	1.6256
P24941	Cyclin-dependent kinase 2	0.0114	1.6367
P61619	Protein transport protein Sec61 subunit alpha isoform 1	0.0398	1.7619
P08195	4F2 cell-surface antigen heavy chain	0.0151	1.7870
Q16881	Thioredoxin reductase 1, cytoplasmic	0.0472	1.8402
P55060	Exportin-2	0.0229	1.9550
P04179	Superoxide dismutase [Mn], mitocondrial	0.0066	2.3668
Q9NVD7	Alpha-parvin	0.0339	2.3802
Q9HDC9	Adipocyte plasma membrane-associated protein	0.0129	2.4386
P62314	Small nuclear ribonucleoprotein Sm D1	0.0349	2.6814
P78344	Eukaryotic translation initiation factor 4 gamma 2	0.0332	10.9652
P07237	Protein disulfide-isomerase	0.0408	1.2058
P78417	Glutathione S-transferase omega-1	0.0425	1.2224
O00299	Chloride intracellular channel protein 1	0.0195	1.2303
P23526	Adenosylhomocysteinase	0.0443	1.2358
Q96S97	Myeloid-associated differentiation marker	0.0496	1.2446
P62258	14-3-3 protein epsilon	0.0079	1.2498
P16152	Carbonyl reductase [NADPH] 1	0.0199	1.2521
P32119	Peroxiredoxin-2	0.0385	1.2540
P00387	NADH-cytochrome b5 reductase 3	0.0087	1.2629
P17655	Calpain-2 catalytic subunit	0.0287	1.2708
P61981	14-3-3 Protein gamma	0.0482	1.2743
P60900	Proteasome subunit alpha type-6	0.0270	1.2756
O43707	Alpha-actinin-4	0.0309	1.2781
P27824	Calnexin	0.0254	1.2798
P11413	Glucose-6-phosphate 1-dehydrogenase	0.0215	1.2906
P08133	Annexin A6	0.0055	1.2937
P12814	Alpha-actinin-1	0.0146	1.3017
P22314	Ubiquitin-like modifier-activating enzyme 1	0.0015	1.3163
P54578	Ubiquitin carboxyl-terminal hydrolase 14	0.0386	1.3214
P49721	Proteasome subunit beta type-2	0.0414	1.3568
Q96AG4	Leucine-rich repeat-containing protein 59	0.0085	1.3642
P67812	Signal peptidase complex catalytic subunit SEC11A	0.0153	1.3671
Q15404	Ras suppressor protein 1	0.0172	1.3808
P07996	Thrombospondin-1	0.0379	1.3992
O00629	Importin subunit alpha-3	0.0386	1.4218
Q9BS26	Endoplasmic reticulum resident protein 44	0.0170	1.4754
Q99798	Aconitate hydratase, mitocondrial	0.0357	1.4799
P60953	Cell division control protein 42 homolog	0.0215	1.4806
Q9Y3I0	tRNA-splicing ligase RtcB homolog	0.0506	1.4807
P2484	Myosin regulatory light polypeptide 9	0.0344	1.5135
Q15758	Neutral amino acid transporter B(0)	0.0076	1.5970
Q13724	Mannosyl-oligosaccharide glucosidase	0.0351	1.6011
P08727	Keratin, type I cytoskeletal 19	0.0315	1.6256
P24941	Cyclin-dependent kinase 2	0.0114	1.6367
P61619	Protein transport protein Sec61 subunit alpha isoform 1	0.0398	1.7619
P08195	4F2 cell-surface antigen heavy chain	0.0151	1.7870
Q16881	Thioredoxin reductase 1, cytoplasmic	0.0472	1.8402
P55060	Exportin-2	0.0229	1.9550
P04179	Superoxide dismutase [Mn], mitocondrial	0.0066	2.3668
Q9NVD7	Alpha-parvin	0.0339	2.3802
Q9HDC9	Adipocyte plasma membrane-associated protein	0.0129	2.4386
P62314	Small nuclear ribonucleoprotein Sm D1	0.0349	2.6814
P78344	Eukaryotic translation initiation factor 4 gamma 2	0.0332	10.9652

FC: fold change.

**Table 3:** Quantitative study of proteins in UMoC vs. MoC with NLC + ERT (A) and vice versa (B).

	**A. Upregulated Proteins in UMoC, Compared in MoC with NLC + ERT**		
**Protein**	**Group**	***p*-Value**	**FC**
P04843	Dolichyl-diphosphooligosaccharide-protein glycosyltransferase subunit 1	0.0416	1.2453
Q7KZF4	Staphylococcal nuclease domain-containing protein 1	0.0308	1.3226
Q99829	Copine-1	0.0098	1.5958
P43686	26S proteasome regulatory subunit 6B	0.0054	1.8527
O75436	Vacuolar protein sorting-associated protein 26A	0.0499	1.2358
P01024	Complement C3	0.0246	2.0811
P02788	Lactotransferrin	0.0130	2.2108
Q9P219	Protein Daple	0.0205	2.3711
P30084	Enoyl-CoA hydratase, mitochondrial	0.0377	2.3790
P19823	Inter-alpha-trypsin inhibitor heavy chain H2	0.0491	2.3791
P00367	Glutamate dehydrogenase 1, mitocondrial	0.0225	2.5313
Q7L1Q6	Basic leucine zipper and W2 domain-containing protein 1	0.0495	2.5351
Q7LBC6	Lysine-specific demethylase 3B	0.0124	2.5605
P02765	Alpha-2-HS-glycoprotein	0.0175	2.6800
**B. Upregulated Proteins in MoC with NLC + ERT, Compared in UMoC**			
**Protein**	**Group**	***p*-Value**	**FC**
P22090	40S ribosomal protein S4, Y isoform 1	0.0134	2.6287
Q9BQB6	Vitamin K epoxide reductase complex subunit 1	0.0413	2.5833
P05783	Keratin, type I cytoskeletal 18	0.0048	2.4465
P51665	26S proteasome non-ATPase regulatory subunit 7	0.0118	2,1633
O75368	SH3 domain-binding glutamic acid-rich-like protein	0.0004	2.0888
Q96AT9	Ribulose-phosphate 3-epimerase	0.0115	2.0690
P48147	Prolyl endopeptidase	0.0276	2.0209
P30498	HLA class I histocompatibility antigen, B-78 alpha chain	0.0315	1.8355
P04179	Superoxide dismutase [Mn], mitochondrial	0.0153	1.8015
Q7Z4H8	KDEL motif-containing protein 2	0.0292	1.7891
Q12797	Aspartyl/asparaginyl beta-hydroxylase	0.0031	1.7517
Q06323	Proteasome activator complex subunit 1	0.0064	1.7109
O15260	Surfeit locus protein 4	0.0346	1.6967
Q86UP2	Kinectin	0.0338	1.6722
P84077	ADP-ribosylation factor 1	0.0069	1.5850
P62857	40S ribosomal protein S28	0.0021	1.5181
P17987	T-complex protein 1 subunit alpha	0.0435	1.5070
P36578	60S ribosomal protein L4	0.0492	1.4908
P09651	Heterogeneous nuclear ribonucleoprotein A1	0.0033	1.4863
O00629	Importin subunit alpha-3	0.0228	1.4728
Q15293	Reticulocalbin-1	0.0212	1.4704
P62805	Histone H4	0.0123	1.4701
P60953	Cell division control protein 42 homolog	0.0168	1.4677
P18669	Phosphoglycerate mutase 1	0.0088	1.4651
P48643	T-complex protein 1 subunit epsilon	0.0003	1.4511
Q99798	Aconitate hydratase, mitochondrial	0.0225	1.4494
P00558	Phosphoglycerate kinase 1	0.0053	1.4426
P60174	Triosephosphate isomerase	0.0062	1.4168
Q16222	UDP-N-acetylhexosamine pyrophosphorylase	0.0459	1.4093
P11021	Endoplasmic reticulum chaperone BiP	0.0005	1.3987
P26599	Polypyrimidine tract-binding protein 1	0.0205	1.3867
Q14152	Eukaryotic translation initiation factor 3 subunit A	0.0272	1.3757
P09936	Ubiquitin carboxyl-terminal hydrolase isozyme L1	0.0402	1.3509
P34897	Serine hydroxymethyltransferase, mitochondrial	0.0384	1.3406
P38646	Stress-70 protein, mitochondrial	0.0023	1.3405
P30101	Protein disulfide-isomerase A3	0.0012	1.3319
P17655	Calpain-2 catalytic subunit	0.0433	1.3318
P07237	Protein disulfide-isomerase	0.0020	1.3316
P18124	60S ribosomal protein L7	0.0196	1.3239
P06733	Alpha-enolase	0.0031	1.3183
P27797	Calreticulin	0.0271	1.3103
Q96AY3	Peptidyl-prolyl cis-trans isomerase FKBP10	0.0103	1.2954
P62269	40S ribosomal protein S18	0.0455	1.2947
Q9NR31	GTP-binding protein SAR1a	0.0097	1.2880
Q13148	TAR DNA-binding protein 43	0.0483	1.2741
P61978	Heterogeneous nuclear ribonucleoprotein K	0.0396	1.2736
P46777	60S ribosomal protein L5	0.0015	1.2716
P62701	40S ribosomal protein S4, X isoform	0.0060	1.2709
P62826	GTP-binding nuclear protein Ran	0.0164	1.2660
P05388	60S acidic ribosomal protein P0	0.0071	1.2656
O95394	Phosphoacetylglucosamine mutase	0.0247	1.2547
O60701	UDP-glucose 6-dehydrogenase	0.0232	1.2538
P62820	Ras-related protein Rab-1A OS=Homo sapiens SV=3	0.0094	1.2514
Q8NBJ5	Procollagen galactosyltransferase 1	0.0389	1.2479
Q9ULV4	Coronin-1C OS=Homo sapiens	0.0338	1.2455
Q14315	Filamin-C OS=Homo sapiens	0.0186	1.2394
Q14697	Neutral alpha-glucosidase AB	0.0435	1.2386
P00338	L-lactate dehydrogenase A chain	0.0260	1.2371
Q6NZI2	Caveolae-associated protein 1	0.0295	1.2301
P07195	L-lactate dehydrogenase B chain	0.0202	1.2247
O00299	Chloride intracellular channel protein 1	0.0501	1.2232
O43707	Alpha-actinin-4	0.0311	1.2167
P12111	Collagen alpha-3(VI) chain	0.0378	1.2093

FC: fold change.

**Table 4:** Data obtained from the Telethon network biogenetic biobank.

Samples	Sex	Diagnosis	Phenotype
S.1 MoC	Male	MPS IVA	Classic
S.2 MoC	Female	MPS IVA	Classic
S.3 MoC	Male	MPS IVA	Classic
S.4 MoC	Female	MPS-IVA	Classic

**Table 5:** Treatment administered in samples.

Samples	Untreated	NLC + ERT	ERT
H.C	X	X	X
S.1 MoC	X	X	
S.2 MoC	X		X
S.3 MoC	X	X	
S.4 MoC	X		X

Note: All the samples were analyzed in triplicate. S. sample; NLC nanostructure lipid carrier; ERT: Enzyme replacement therapy.

**Table 6:** Values of enzyme activity.

Samples	Before Treatment (nM/h/mg)	After Treatment (nM/h/mg)
S.1 MoC	0.0	1.6
S.2 MoC	0.0	4.8
S.3 MoC	0.0	6.5
S.4 MoC	0.0	8.9

## Supplementary Materials

Supplementary materials can be found at https://www.mdpi.com/1422-0067/20/18/4610/s1.

## Abbreviations

C6S	Chondroitin 6 sulphate
ERT	Enzyme replacement therapy
FC	Fold change
GAG	Glycosaminoglycan
GALNS	N-acetylgalactosamine-6-sulfatase
KS	Keratan sulphateJ
LC	Liquid chromatography
MPS IVA	Mucopolysaccharidosis type IVA
MRM	Multiple reaction monitoring
MS	Mass spectrometry
NLC	Nanostructure lipid carrier
S	Sample
SDC	Sodium dodecyl sulfate
SRM	Selected reaction monitoring
SNARE	soluble N-ethylmaleimide-sensitive factor attachment protein receptor
SWATH-MS	Sequential window acquisition of all theoretical mass spectra
UMoC	Untreated Morquio A cells
