# Supplementary material for: Proteomic Analysis in Morquio A Cells Treated with Immobilized Enzymatic Replacement Therapy on Nanostructured Lipid Systems
Source: Int J Mol Sci. 2019 Sep 18;20(18):4610. doi: 10.3390/ijms20184610 (PMC6769449; doi:10.3390/ijms20184610)
Supplement: Supplementary file 1 [file ijms-20-04610-s001.zip › Supplementary Table 1.docx]

| **Proteins library pathological cells Vs Proteins pathological cells +ERT** | | | | | |
| --- | --- | --- | --- | --- | --- |
| Protein | Group | | p-value | | Fold Change |
| P16435 | NADPH--cytochrome P450 reductase | | 0,1877 | | 6,2972 |
| P29966 | Myristoylatedalanine-rich C-kinasesubstrate | | 0,0963 | | 5,2407 |
| P54886 | Delta-1-pyrroline-5-carboxylate synthase | | 0,1171 | | 3,6476 |
| Q9NQC3 | Reticulon-4 | | 0,0522 | | 3,5220 |
| P05198 | Eukaryotictranslationinitiation factor 2 subunit 1 | | 0,1750 | | 2,9081 |
| P29373 | Cellularretinoicacid-bindingprotein 2 | | 0,1435 | | 2,7313 |
| Q07955 | Serine/arginine-richsplicing factor 1 | | 0,2391 | | 2,6153 |
| P22090 | 40S ribosomal protein S4, Y isoform 1 | | 0,1397 | | 2,4831 |
| Q13620 | Cullin-4B | | 0,3466 | | 2,4074 |
| P52306 | Rap1 GTPase-GDP dissociationstimulator 1 | | 0,4653 | | 2,3323 |
| P42566 | Epidermalgrowth factor receptor substrate 15 | | 0,2963 | | 2,3238 |
| P33992 | DNA replicationlicensing factor MCM5 | | 0,4452 | | 2,0958 |
| Q9UHG3 | Prenylcysteine oxidase 1 | | 0,0935 | | 2,0515 |
| P27816 | Microtubule-associatedprotein 4 | | 0,4871 | | 2,0243 |
| P02647 | Apolipoprotein A-I | | 0,0642 | | 2,0028 |
| P61247 | 40S ribosomal protein S3a | | 0,1473 | | 2,0023 |
| Q9H4A4 | Aminopeptidase B | | 0,0813 | | 2,0007 |
| O95479 | GDH/6PGL endoplasmicbifunctionalprotein | | 0,0517 | | 1,9883 |
| P19367 | Hexokinase-1 | | 0,4603 | | 1,9744 |
| Q13151 | Heterogeneous nuclear ribonucleoprotein A0 | | 0,0660 | | 1,8960 |
| P46776 | 60S ribosomal protein L27a | | 0,0605 | | 1,8654 |
| P17844 | Probable ATP-dependent RNA helicase DDX5 | | 0,1045 | | 1,8618 |
| Q3SY69 | Mitochondrial 10-formyltetrahydrofolate dehydrogenase | | 0,0645 | | 1,8612 |
| P09622 | Dihydrolipoyldehydrogenase, mitochondrial | | 0,0977 | | 1,8456 |
| Q92841 | Probable ATP-dependent RNA helicase DDX17 | | 0,1232 | | 1,8393 |
| P06702 | Protein S100-A9 | | 0,1894 | | 1,8110 |
| P46779 | 60S ribosomal protein L28 | | 0,3459 | | 1,7800 |
| O60763 | General vesicular transport factor p115 | | 0,1141 | | 1,7749 |
| P62750 | 60S ribosomal protein L23a | | 0,1221 | | 1,7192 |
| Q03252 | Lamin-B2 | | 0,3437 | | 1,6878 |
| P48739 | Phosphatidylinositol transfer protein beta isoform | | 0,2549 | | 1,6601 |
| P62081 | 40S ribosomal protein S7 | | 0,1263 | | 1,6424 |
| P43487 | Ran-specificGTPase-activatingprotein | | 0,1448 | | 1,6341 |
| P62241 | 40S ribosomal protein S8 | | 0,0846 | | 1,6293 |
| Q6PEY2 | Tubulin alpha-3E chain | | 0,2470 | | 1,6281 |
| P16402 | Histone H1.3 | | 0,2442 | | 1,6118 |
| P62280 | 40S ribosomal protein S11 | | 0,2534 | | 1,6045 |
| Q92945 | Far upstreamelement-bindingprotein 2 | | 0,0956 | | 1,6006 |
| P12110 | Collagen alpha-2(VI) chain | | 0,0762 | | 1,5830 |
| P60033 | CD81 antigen | | 0,2964 | | 1,5769 |
| P68371 | Tubulin beta-4B chain | | 0,0867 | | 1,5758 |
| Q13045 | Protein flightless-1 homolog | | 0,4165 | | 1,5671 |
| P61604 | 10 kDaheat shock protein, mitochondrial | | 0,1690 | | 1,5670 |
| Q9BXS5 | AP-1 complexsubunit mu-1 | | 0,1465 | | 1,5622 |
| P61221 | ATP-bindingcassettesub-family E member 1 | | 0,6457 | | 1,5415 |
| Q15102 | Platelet-activating factor acetylhydrolase IB subunit gamma | | 0,4525 | | 1,5359 |
| O95816 | BAG family molecular chaperoneregulator 2 | | 0,1150 | | 1,5184 |
| Q04828 | Aldo-ketoreductasefamily 1 member C1 | | 0,3815 | | 1,5181 |
| P16083 | Ribosyldihydronicotinamidedehydrogenase [quinone] | | 0,3291 | | 1,4753 |
| P60866 | 40S ribosomal protein S20 | | 0,1641 | | 1,4736 |
| P62277 | 40S ribosomal protein S13 | | 0,2684 | | 1,4669 |
| P10253 | Lysosomalalpha-glucosidase | | 0,1393 | | 1,4650 |
| Q96FB5 | Protein RRNAD1 | | 0,6338 | | 1,4620 |
| Q9NYU2 | UDP-glucose:glycoproteinglucosyltransferase 1 | | 0,3417 | | 1,4559 |
| P40429 | 60S ribosomal protein L13a | | 0,1843 | | 1,4549 |
| P52597 | Heterogeneous nuclear ribonucleoprotein F | | 0,3964 | | 1,4504 |
| Q9P0K7 | Ankycorbin | | 0,3529 | | 1,4410 |
| P51571 | Translocon-associatedproteinsubunit delta | | 0,2351 | | 1,4182 |
| Q7L576 | Cytoplasmic FMR1-interacting protein 1 | | 0,1000 | | 1,4098 |
| P00738 | Haptoglobin | | 0,4767 | | 1,3993 |
| P50454 | Serpin H1 | | 0,1211 | | 1,3906 |
| P02452 | Collagen alpha-1(I) chain | | 0,1999 | | 1,3854 |
| P23634 | Plasma membranecalcium-transportingATPase 4 | | 0,2410 | | 1,3727 |
| P02765 | Alpha-2-HS-glycoprotein | | 0,4054 | | 1,3631 |
| P68871 | Hemoglobinsubunit beta | | 0,7055 | | 1,3625 |
| Q15631 | Translin | | 0,6435 | | 1,3571 |
| Q96D15 | Reticulocalbin-3 | | 0,3147 | | 1,3470 |
| P13647 | Keratin, type II cytoskeletal 5 | | 0,5485 | | 1,3424 |
| Q10567 | AP-1 complexsubunit beta-1 | | 0,5007 | | 1,3317 |
| Q15393 | Splicing factor 3B subunit 3 | | 0,3044 | | 1,3280 |
| P46782 | 40S ribosomal protein S5 | | 0,4731 | | 1,3273 |
| Q9NZN4 | EH domain-containingprotein 2 | | 0,1247 | | 1,3233 |
| P62333 | 26S proteasomeregulatorysubunit 10B | | 0,2375 | | 1,3082 |
| Q09666 | Neuroblastdifferentiation-associatedprotein AHNAK | | 0,3786 | | 1,3012 |
| P61353 | 60S ribosomal protein L27 | | 0,2387 | | 1,3008 |
| P51688 | N-sulphoglucosaminesulphohydrolase OS=Homo sapiens | | 0,2836 | | 1,2901 |
| P09936 | Ubiquitincarboxyl-terminal hydrolaseisozyme L1 | | 0,1722 | | 1,2875 |
| P01871 | Immunoglobulin heavy constant mu | | 0,6020 | | 1,2869 |
| Q16822 | Phosphoenolpyruvatecarboxykinase [GTP], mitochondrial | | 0,3548 | | 1,2840 |
| P54920 | Alpha-soluble NSF attachmentprotein | | 0,3601 | | 1,2812 |
| P62195 | 26S proteasomeregulatorysubunit 8 | | 0,4705 | | 1,2732 |
| P41091 | Eukaryotictranslationinitiation factor 2 subunit 3 | | 0,3879 | | 1,2731 |
| P62913 | 60S ribosomal protein L11 | | 0,4379 | | 1,2676 |
| P98082 | Disabledhomolog 2 | | 0,1764 | | 1,2671 |
| P69905 | Hemoglobinsubunitalpha | | 0,7525 | | 1,2620 |
| P39023 | 60S ribosomal protein L3 | | 0,1829 | | 1,2614 |
| P48637 | Glutathionesynthetase | | 0,3824 | | 1,2595 |
| P37108 | Signalrecognitionparticle 14 kDaprotein | | 0,4677 | | 1,2593 |
| P16070 | CD44 antigen | | 0,2865 | | 1,2574 |
| P08123 | Collagen alpha-2(I) chain | | 0,3801 | | 1,2510 |
| Q14103 | Heterogeneous nuclear ribonucleoprotein D0 | | 0,0997 | | 1,2480 |
| P05556 | Integrin beta-1 | | 0,2637 | | 1,2449 |
| P68363 | Tubulin alpha-1B chain | | 0,3742 | | 1,2443 |
| O60664 | Perilipin-3 | | 0,1810 | | 1,2376 |
| P17858 | ATP-dependent 6-phosphofructokinase, livertype | | 0,3591 | | 1,2334 |
| Q15417 | Calponin-3 | | 0,5848 | | 1,2320 |
| P02533 | Keratin, type I cytoskeletal 14 | | 0,7410 | | 1,2308 |
| O43399 | Tumor protein D54 | | 0,4105 | | 1,2291 |
| O00303 | Eukaryotictranslationinitiation factor 3 subunit F | | 0,4733 | | 1,2290 |
| P15311 | Ezrin | | 0,4718 | | 1,2287 |
| P36578 | 60S ribosomal protein L4 | | 0,3074 | | 1,2287 |
| P21281 | V-typeprotonATPasesubunit B, brainisoform | | 0,2389 | | 1,2250 |
| P62424 | 60S ribosomal protein L7a | | 0,2194 | | 1,2221 |
| P25705 | ATP synthasesubunitalpha, mitochondrial | | 0,3058 | | 1,2194 |
| Q99877 | Histone H2B type 1-N | | 0,2910 | | 1,2173 |
| P62269 | 40S ribosomal protein S18 | | 0,2242 | | 1,2156 |
| P19338 | Nucleolin | | 0,0535 | | 1,2146 |
| P12111 | Collagen alpha-3(VI) chain | | 0,1195 | | 1,2144 |
| P49748 | Verylong-chainspecificacyl-CoAdehydrogenase, mitochondrial | | 0,4224 | | 1,2140 |
| Q9Y3F4 | Serine-threoninekinase receptor-associatedprotein | | 0,5266 | | 1,2115 |
| P62244 | 40S ribosomal protein S15a | | 0,3986 | | 1,2094 |
| P62249 | 40S ribosomal protein S16 | | 0,1237 | | 1,2072 |
| P00367 | Glutamatedehydrogenase 1, mitochondrial | | 0,4925 | | 1,2030 |
| P50914 | 60S ribosomal protein L14 | | 0,5449 | | 1,2029 |
| **Proteinslibrerycellspathologhical + NLC+ERT Vs Proteinspathologicalcells** | | | | | |
| Protein | | Group | | p-value | Fold Change |
| Q9Y262 | | Eukaryotictranslationinitiation factor 3 subunit L | | 0,2302 | 1,2015 |
| P18206 | | Vinculin | | 0,0591 | 1,2025 |
| P11142 | | Heat shock cognate 71 kDaprotein | | 0,0951 | 1,2042 |
| P78371 | | T-complexprotein 1 subunit beta | | 0,1539 | 1,2066 |
| P23284 | | Peptidyl-prolylcis-trans isomerase B | | 0,1077 | 1,2093 |
| Q9BRA2 | | Thioredoxindomain-containingprotein 17 | | 0,4036 | 1,2095 |
| P52907 | | F-actin-cappingproteinsubunit alpha-1 | | 0,1491 | 1,2105 |
| P14618 | | Pyruvatekinase PKM | | 0,0734 | 1,2116 |
| P62854 | | 40S ribosomal protein S26 | | 0,6093 | 1,2125 |
| Q99832 | | T-complexprotein 1 subunit eta | | 0,4176 | 1,2126 |
| P12110 | | Collagen alpha-2(VI) chain | | 0,3468 | 1,2135 |
| O14617 | | AP-3 complexsubunit delta-1 | | 0,2629 | 1,2149 |
| P63241 | | Eukaryotictranslationinitiation factor 5A-1 | | 0,4717 | 1,2164 |
| Q12797 | | Aspartyl/asparaginyl beta-hydroxylase | | 0,4096 | 1,2176 |
| Q13637 | | Ras-relatedprotein Rab-32 | | 0,5200 | 1,2187 |
| P49591 | | Serine--tRNA ligase, cytoplasmic | | 0,3143 | 1,2188 |
| O60506 | | Heterogeneous nuclear ribonucleoprotein Q | | 0,0773 | 1,2202 |
| P68363 | | Tubulin alpha-1B chain | | 0,4595 | 1,2217 |
| P47756 | | F-actin-cappingproteinsubunit beta | | 0,1329 | 1,2218 |
| Q12792 | | Twinfilin-1 | | 0,3442 | 1,2223 |
| P02511 | | Alpha-crystallin B chain | | 0,4835 | 1,2233 |
| P50454 | | Serpin H1 | | 0,3612 | 1,2283 |
| Q9UI10 | | Translationinitiation factor eIF-2B subunit delta | | 0,6176 | 1,2287 |
| P61981 | | 14-3-3 protein gamma | | 0,1833 | 1,2296 |
| Q00341 | | Vigilin | | 0,3912 | 1,2310 |
| P12955 | | Xaa-Pro dipeptidase | | 0,1916 | 1,2320 |
| Q9BZF9 | | Uveal autoantigenwithcoiled-coildomains and ankyrinrepeats | | 0,5939 | 1,2352 |
| P52565 | | Rho GDP-dissociationinhibitor 1 | | 0,1822 | 1,2391 |
| Q14204 | | Cytoplasmicdynein 1 heavy chain 1 | | 0,1696 | 1,2391 |
| P84077 | | ADP-ribosylation factor 1 | | 0,2562 | 1,2393 |
| P32322 | | Pyrroline-5-carboxylate reductase 1, mitochondrial | | 0,1480 | 1,2394 |
| O95479 | | GDH/6PGL endoplasmicbifunctionalprotein | | 0,4190 | 1,2397 |
| P31946 | | 14-3-3 protein beta/alpha | | 0,1574 | 1,2416 |
| Q15404 | | Ras suppressorprotein 1 | | 0,2270 | 1,2418 |
| O43175 | | D-3-phosphoglycerate dehydrogenase | | 0,1346 | 1,2432 |
| Q16555 | | Dihydropyrimidinase-relatedprotein 2 | | 0,1645 | 1,2442 |
| Q99614 | | Tetratricopeptiderepeatprotein 1 | | 0,6643 | 1,2459 |
| Q96AY3 | | Peptidyl-prolylcis-trans isomerase FKBP10 | | 0,1045 | 1,2464 |
| P34897 | | Serinehydroxymethyltransferase, mitochondrial | | 0,2271 | 1,2480 |
| O75367 | | Core histone macro-H2A.1 | | 0,4565 | 1,2480 |
| P07741 | | Adeninephosphoribosyltransferase | | 0,2661 | 1,2491 |
| P13797 | | Plastin-3 | | 0,0879 | 1,2516 |
| P78417 | | Glutathione S-transferase omega-1 | | 0,0606 | 1,2520 |
| O94925 | | Glutaminasekidneyisoform, mitochondrial | | 0,4142 | 1,2544 |
| O75083 | | WD repeat-containingprotein 1 | | 0,0908 | 1,2572 |
| P25705 | | ATP synthasesubunitalpha, mitochondrial | | 0,2702 | 1,2585 |
| P07355 | | Annexin A2 | | 0,0520 | 1,2585 |
| P68036 | | Ubiquitin-conjugatingenzyme E2 L3 | | 0,0523 | 1,2596 |
| O00410 | | Importin-5 | | 0,1720 | 1,2602 |
| P22626 | | Heterogeneous nuclear ribonucleoproteins A2/B1 | | 0,0722 | 1,2607 |
| P10599 | | Thioredoxin | | 0,2691 | 1,2622 |
| P30048 | | Thioredoxin-dependentperoxidereductase, mitochondrial | | 0,3634 | 1,2631 |
| Q04917 | | 14-3-3 protein eta | | 0,0768 | 1,2644 |
| P60842 | | Eukaryoticinitiation factor 4A-I | | 0,4544 | 1,2655 |
| P26641 | | Elongation factor 1-gamma | | 0,0758 | 1,2676 |
| Q7L576 | | Cytoplasmic FMR1-interacting protein 1 | | 0,2090 | 1,2700 |
| P63151 | | Serine/threonine-proteinphosphatase 2A 55 kDaregulatorysubunit B alphaisoform | | 0,5656 | 1,2708 |
| Q92520 | | Protein FAM3C | | 0,3718 | 1,2716 |
| Q6IBS0 | | Twinfilin-2 | | 0,2171 | 1,2739 |
| Q12906 | | Interleukinenhancer-binding factor 3 | | 0,4265 | 1,2741 |
| P62269 | | 40S ribosomal protein S18 | | 0,0995 | 1,2783 |
| Q00610 | | Clathrin heavy chain 1 | | 0,0740 | 1,2821 |
| P46776 | | 60S ribosomal protein L27a | | 0,3967 | 1,2878 |
| Q13148 | | TAR DNA-bindingprotein 43 | | 0,0830 | 1,2878 |
| P09104 | | Gamma-enolase | | 0,5702 | 1,2883 |
| Q15008 | | 26S proteasome non-ATPaseregulatorysubunit 6 | | 0,0587 | 1,2899 |
| Q1KMD3 | | Heterogeneous nuclear ribonucleoprotein U-likeprotein 2 | | 0,0505 | 1,2928 |
| Q16181 | | Septin-7 | | 0,2062 | 1,2958 |
| P54577 | | Tyrosine--tRNA ligase, cytoplasmic | | 0,0590 | 1,2969 |
| P17655 | | Calpain-2 catalyticsubunit | | 0,0543 | 1,2992 |
| P41240 | | Tyrosine-proteinkinase CSK | | 0,2608 | 1,2997 |
| P84103 | | Serine/arginine-richsplicing factor 3 | | 0,0589 | 1,3031 |
| P60953 | | Cell division control protein 42 homolog | | 0,2211 | 1,3031 |
| Q07866 | | Kinesin light chain 1 | | 0,5372 | 1,3053 |
| Q01995 | | Transgelin | | 0,2459 | 1,3104 |
| P13804 | | Electron transfer flavoproteinsubunitalpha, mitochondrial | | 0,1753 | 1,3134 |
| Q99873 | | Proteinarginine N-methyltransferase 1 | | 0,0974 | 1,3139 |
| P35222 | | Catenin beta-1 | | 0,3872 | 1,3153 |
| Q13510 | | Acidceramidase | | 0,2768 | 1,3180 |
| A0FGR8 | | Extended synaptotagmin-2 | | 0,2955 | 1,3195 |
| P33778 | | Histone H2B type 1-B | | 0,1099 | 1,3236 |
| Q8WX93 | | Palladin | | 0,4497 | 1,3254 |
| P35232 | | Prohibitin | | 0,5010 | 1,3263 |
| P41091 | | Eukaryotictranslationinitiation factor 2 subunit 3 | | 0,3634 | 1,3266 |
| Q13151 | | Heterogeneous nuclear ribonucleoprotein A0 | | 0,2157 | 1,3270 |
| Q14192 | | Four and a half LIM domainsprotein 2 | | 0,1945 | 1,3326 |
| Q06210 | | Glutamine--fructose-6-phosphate aminotransferase [isomerizing] 1 | | 0,2686 | 1,3347 |
| Q9NYU2 | | UDP-glucose:glycoproteinglucosyltransferase 1 | | 0,5009 | 1,3381 |
| P24844 | | Myosinregulatory light polypeptide 9 | | 0,2962 | 1,3384 |
| Q99798 | | Aconitatehydratase, mitochondrial | | 0,2277 | 1,3431 |
| Q92841 | | Probable ATP-dependent RNA helicase DDX17 | | 0,3451 | 1,3514 |
| Q04637 | | Eukaryotictranslationinitiation factor 4 gamma 1 | | 0,3280 | 1,3519 |
| O60684 | | Importinsubunit alpha-7 | | 0,6090 | 1,3593 |
| P60866 | | 40S ribosomal protein S20 | | 0,2574 | 1,3600 |
| P46783 | | 40S ribosomal protein S10 | | 0,2488 | 1,3655 |
| O14880 | | Microsomalglutathione S-transferase 3 | | 0,3078 | 1,3734 |
| Q9HC38 | | Glyoxalasedomain-containingprotein 4 | | 0,2621 | 1,3786 |
| P42704 | | Leucine-rich PPR motif-containingprotein, mitochondrial | | 0,2871 | 1,3792 |
| P20618 | | Proteasomesubunit beta type-1 | | 0,1378 | 1,3792 |
| Q9UBS4 | | DnaJhomologsubfamily B member 11 | | 0,0592 | 1,3895 |
| Q9P2J5 | | Leucine--tRNA ligase, cytoplasmic | | 0,0991 | 1,3926 |
| Q12904 | | AminoacyltRNAsynthasecomplex-interactingmultifunctionalprotein 1 | | 0,4017 | 1,3940 |
| P40926 | | Malatedehydrogenase, mitochondrial | | 0,1056 | 1,3942 |
| Q86YQ8 | | Copine-8 | | 0,0874 | 1,3973 |
| P62873 | | Guaninenucleotide-bindingprotein G(I)/G(S)/G(T) subunit beta-1 | | 0,0968 | 1,3984 |
| P62805 | | Histone H4 | | 0,0882 | 1,4068 |
| O00303 | | Eukaryotictranslationinitiation factor 3 subunit F | | 0,1254 | 1,4098 |
| P25789 | | Proteasomesubunitalpha type-4 | | 0,1316 | 1,4114 |
| P17987 | | T-complexprotein 1 subunitalpha | | 0,1560 | 1,4134 |
| P26447 | | Protein S100-A4 | | 0,2998 | 1,4213 |
| P07996 | | Thrombospondin-1 | | 0,1020 | 1,4237 |
| P14866 | | Heterogeneous nuclear ribonucleoprotein L | | 0,1442 | 1,4299 |
| P10768 | | S-formylglutathionehydrolase | | 0,2765 | 1,4306 |
| P31949 | | Protein S100-A11 | | 0,1631 | 1,4312 |
| P30046 | | D-dopachromedecarboxylase | | 0,1833 | 1,4338 |
| Q8IVL6 | | Prolyl 3-hydroxylase 3 | | 0,3969 | 1,4385 |
| P16070 | | CD44 antigen | | 0,0594 | 1,4395 |
| Q96HE7 | | ERO1-like proteinalpha | | 0,2814 | 1,4423 |
| Q99877 | | Histone H2B type 1-N | | 0,0723 | 1,4449 |
| P08727 | | Keratin, type I cytoskeletal 19 | | 0,1946 | 1,4522 |
| O75368 | | SH3 domain-bindingglutamicacid-rich-likeprotein | | 0,1195 | 1,4616 |
| Q9P0K7 | | Ankycorbin | | 0,1547 | 1,4631 |
| Q9ULZ3 | | Apoptosis-associatedspeck-likeproteincontaining a CARD | | 0,1332 | 1,4639 |
| Q13596 | | Sorting nexin-1 | | 0,3389 | 1,4655 |
| P00505 | | Aspartateaminotransferase, mitochondrial | | 0,1189 | 1,4663 |
| P02774 | | Vitamin D-bindingprotein | | 0,3962 | 1,4768 |
| O75915 | | PRA1 familyprotein 3 | | 0,0794 | 1,4875 |
| Q96TA1 | | Niban-likeprotein 1 | | 0,0559 | 1,5001 |
| Q9Y230 | | RuvB-like 2 | | 0,2507 | 1,5005 |
| Q9UJZ1 | | Stomatin-likeprotein 2, mitochondrial | | 0,1047 | 1,5030 |
| P40261 | | Nicotinamide N-methyltransferase | | 0,1109 | 1,5045 |
| P11047 | | Lamininsubunit gamma-1 | | 0,2380 | 1,5057 |
| P10809 | | 60 kDaheat shock protein, mitochondrial | | 0,0797 | 1,5137 |
| P62191 | | 26S proteasomeregulatorysubunit 4 | | 0,1299 | 1,5138 |
| O75390 | | Citratesynthase, mitochondrial | | 0,1221 | 1,5148 |
| P20337 | | Ras-relatedprotein Rab-3B | | 0,2209 | 1,5166 |
| P61163 | | Alpha-centractin | | 0,1357 | 1,5286 |
| P60228 | | Eukaryotictranslationinitiation factor 3 subunit E | | 0,1114 | 1,5288 |
| P36578 | | 60S ribosomal protein L4 | | 0,0532 | 1,5316 |
| Q96AT9 | | Ribulose-phosphate 3-epimerase | | 0,3322 | 1,5472 |
| P61247 | | 40S ribosomal protein S3a | | 0,2363 | 1,5594 |
| P56537 | | Eukaryotictranslationinitiation factor 6 | | 0,0775 | 1,5627 |
| P53634 | | Dipeptidylpeptidase 1 | | 0,4760 | 1,5704 |
| Q92900 | | Regulatorofnonsensetranscripts 1 | | 0,0873 | 1,5781 |
| Q9Y3I0 | | tRNA-splicing ligase RtcBhomolog | | 0,0860 | 1,5849 |
| P00738 | | Haptoglobin | | 0,2781 | 1,5963 |
| P62195 | | 26S proteasomeregulatorysubunit 8 | | 0,1503 | 1,6027 |
| Q9UL25 | | Ras-relatedprotein Rab-21 | | 0,0833 | 1,6123 |
| P54920 | | Alpha-soluble NSF attachmentprotein | | 0,0547 | 1,6174 |
| P21964 | | Catechol O-methyltransferase | | 0,1471 | 1,6219 |
| P51571 | | Translocon-associatedproteinsubunit delta | | 0,0558 | 1,6232 |
| Q92734 | | Protein TFG | | 0,1437 | 1,6564 |
| P48739 | | Phosphatidylinositol transfer protein beta isoform | | 0,1979 | 1,6701 |
| Q6NUM9 | | All-trans-retinol 13,14-reductase | | 0,0666 | 1,7014 |
| Q9HDC9 | | Adipocyte plasma membrane-associatedprotein | | 0,2677 | 1,7058 |
| O75347 | | Tubulin-specificchaperone A | | 0,2270 | 1,7105 |
| O00754 | | Lysosomalalpha-mannosidase | | 0,1332 | 1,7143 |
| P48147 | | Prolylendopeptidase | | 0,1126 | 1,7317 |
| O95573 | | Long-chain-fatty-acid--CoA ligase 3 | | 0,1681 | 1,7578 |
| P46782 | | 40S ribosomal protein S5 | | 0,1011 | 1,7853 |
| P22087 | | rRNA 2'-O-methyltransferase fibrillarin | | 0,0986 | 1,7905 |
| P52272 | | Heterogeneous nuclear ribonucleoprotein M | | 0,0858 | 1,7961 |
| P0DP25 | | Calmodulin-3 | | 0,0999 | 1,8272 |
| P60033 | | CD81 antigen | | 0,0927 | 1,8408 |
| P20929 | | Nebulin | | 0,1839 | 1,8547 |
| P62280 | | 40S ribosomal protein S11 | | 0,1067 | 1,9001 |
| P49189 | | 4-trimethylaminobutyraldehyde dehydrogenase | | 0,3644 | 1,9252 |
| P67775 | | Serine/threonine-proteinphosphatase 2A catalyticsubunitalphaisoform | | 0,0562 | 2,1117 |
| P11177 | | Pyruvatedehydrogenase E1 componentsubunit beta, mitochondrial | | 0,1985 | 2,1510 |
| Q96FB5 | | Protein RRNAD1 | | 0,1636 | 2,5340 |
| P19367 | | Hexokinase-1 | | 0,1728 | 2,6654 |
