# Supplementary figures and images for: Proteomic Analysis in Morquio A Cells Treated with Immobilized Enzymatic Replacement Therapy on Nanostructured Lipid Systems
Source: Int J Mol Sci. 2019 Sep 18;20(18):4610. doi: 10.3390/ijms20184610 (PMC6769449; doi:10.3390/ijms20184610)

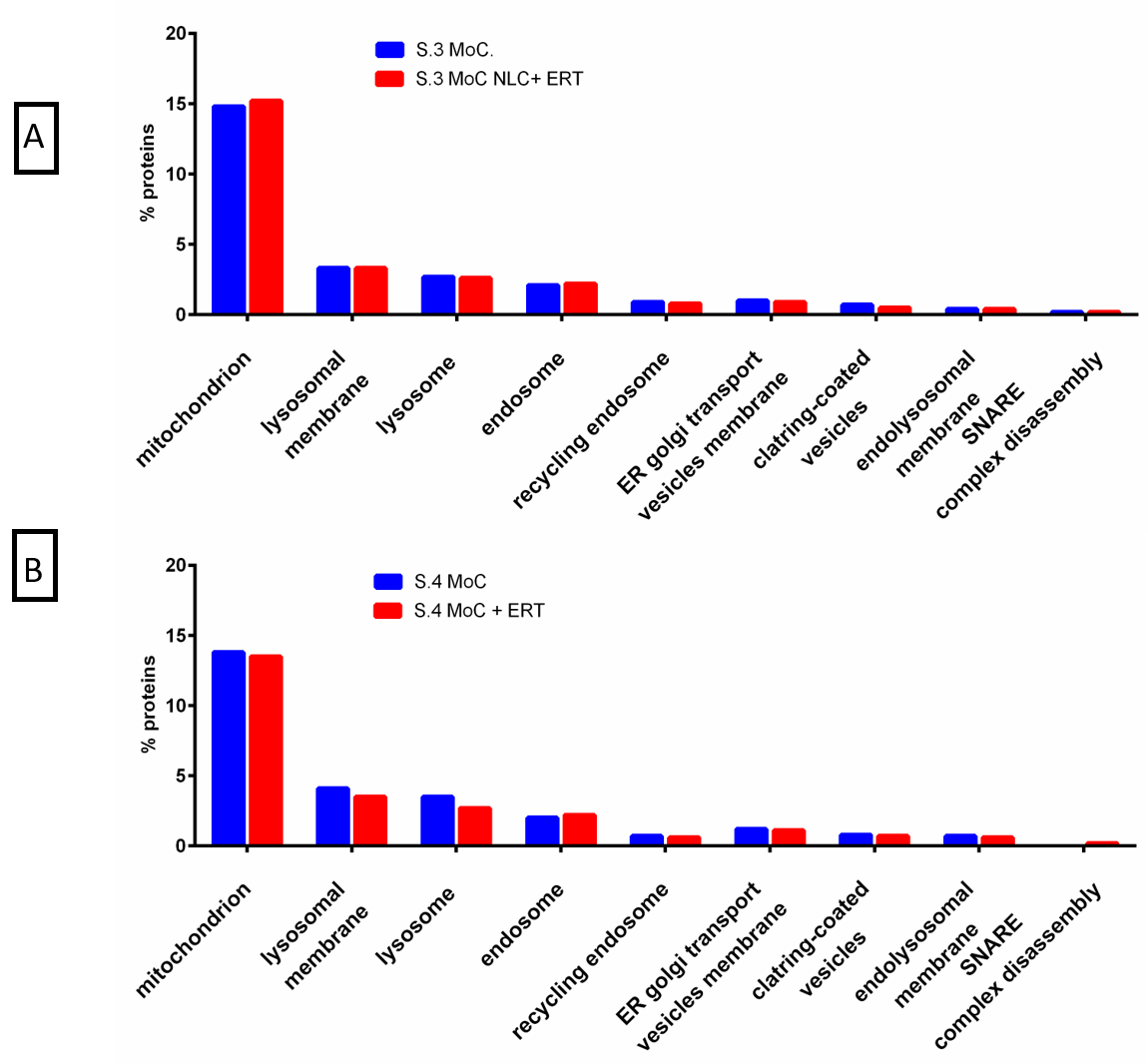

Supplement: Supplementary file 1 [file ijms-20-04610-s001.zip › suplementary figure 2.tif]

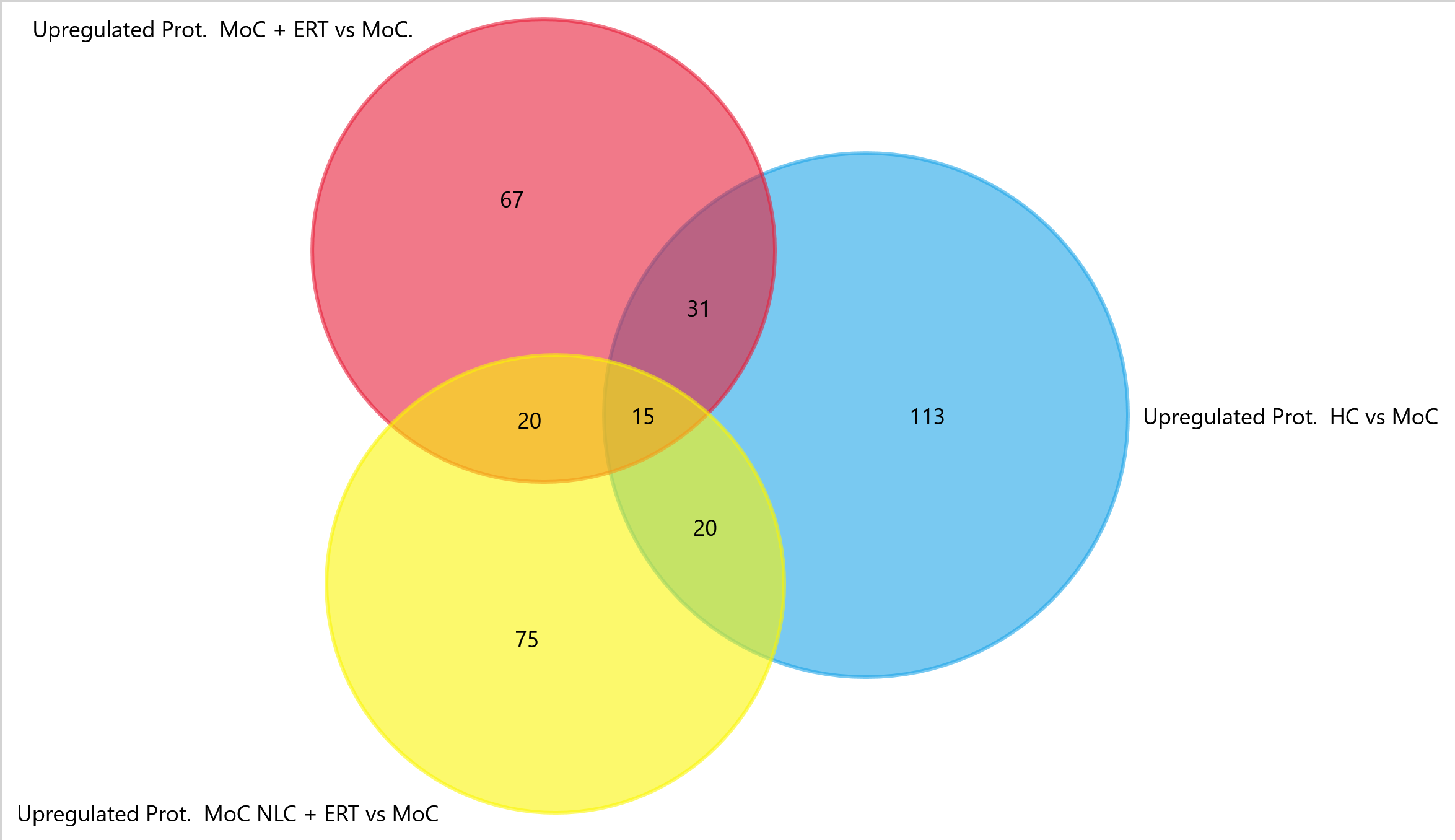

Supplement: Supplementary file 1 [file ijms-20-04610-s001.zip › supplymentary figure 3.tif]

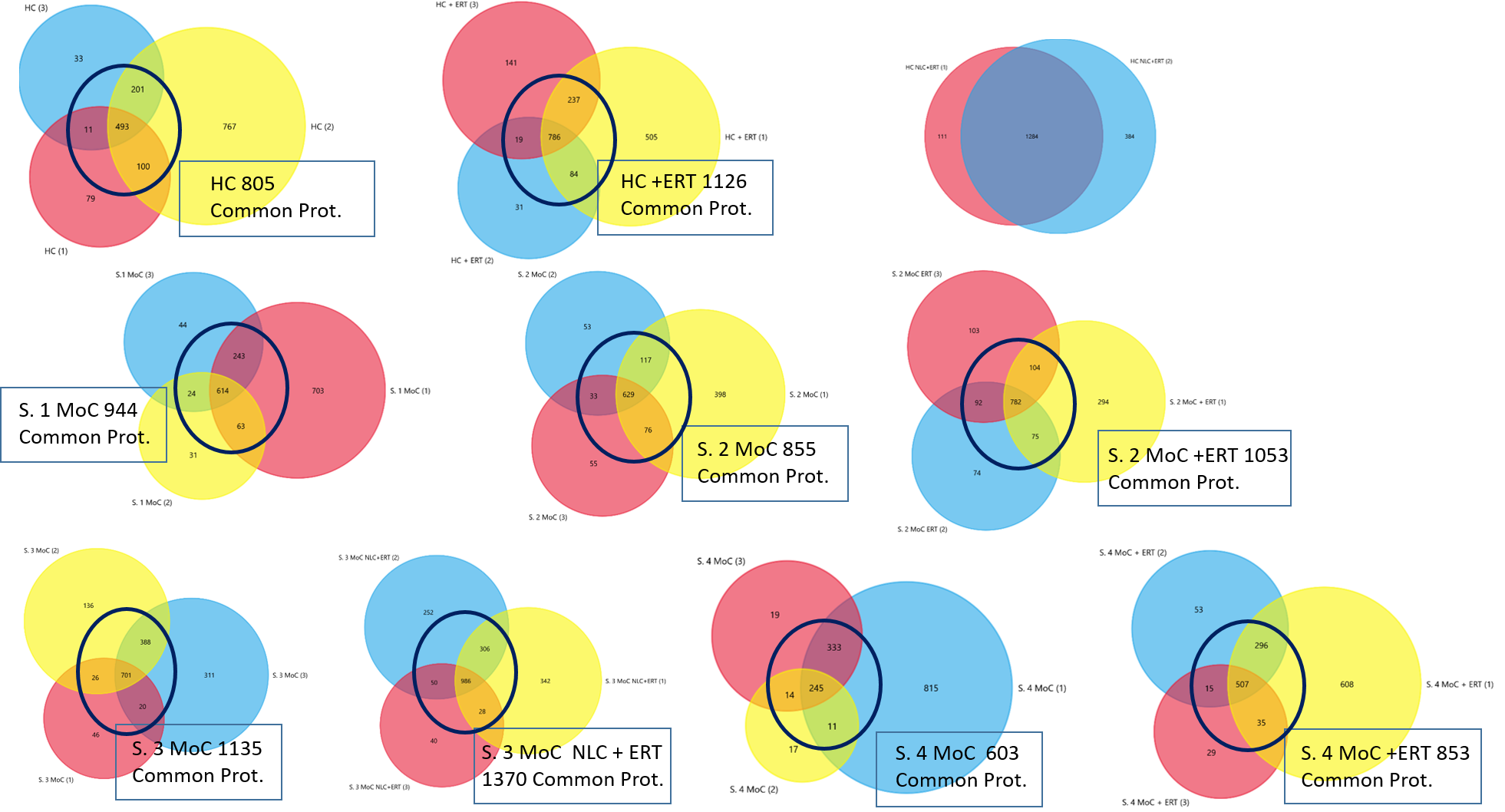

Supplement: Supplementary file 1 [file ijms-20-04610-s001.zip › supplementary figure 1.tif]
